# The Tomato Leucine-Rich Repeat Receptor-Like Kinases *SlSERK3A* and *SlSERK3B* Have Overlapping Functions in Bacterial and Nematode Innate Immunity

**DOI:** 10.1371/journal.pone.0093302

**Published:** 2014-03-27

**Authors:** Hsuan-Chieh Peng, Isgouhi Kaloshian

**Affiliations:** Department of Nematology, Graduate Program in Botany and Plant Sciences, Center for Plant Cell Biology, University of California Riverside, Riverside, California, United States of America; James Hutton Institute, United Kingdom

## Abstract

The Somatic Embryogenesis Receptor Kinase 3 (SERK3)/Brassinosteroid (BR) Insensitive 1-Associated Kinase 1 (BAK1) is required for pattern-triggered immunity (PTI) in *Arabidopsis thaliana* and *Nicotiana benthamiana*. Tomato (*Solanum lycopersicum*) has three *Sl*SERK members. Two of them exhibit particularly high levels of sequence similarity to *At*SERK3 and, therefore, were named *Sl*SERK3A and *Sl*SERK3B. To characterize a role for *SlSERK3A* and *SlSERK3B* in defense, we suppressed each gene individually or co-silenced both using virus-induced gene silencing (VIGS) in the tomato cv. Moneymaker. Co-silencing *SlSERK3A* and *SlSERK3B* resulted in spontaneous necrotic lesions and reduced sensitivity to exogenous BR treatment. Silencing either *SlSERK3A* or *SlSERK3B* resulted in enhanced susceptibility to root knot-nematode and to non-pathogenic *Pseudomonas syringae* pv. *tomato (Pst)* DC3000 *hrcC* indicating that both *SlSERK3*s are positive regulators of defense. Interestingly, silencing *SlSERK3B*, but not *SlSERK3A*, resulted in enhanced susceptibility to the pathogenic strain *Pst* DC3000 indicating distinct roles for these two *SlSERK3* paralogs. *Sl*SERK3A and *Sl*SERK3B are active kinases, localized to the plasma membrane, and interact *in vivo* with the Flagellin Sensing 2 receptor in a flg22-dependent manner. Complementation of the *Atserk3/bak1-4* mutant with either *SlSERK3A* or *SlSERK3B* partially rescued the mutant phenotype. Thus, *SlSERK3A* and *SlSERK3B* are likely to constitute tomato orthologs of *BAK1*.

## Introduction

Innate immunity is the genetically determined and inheritable ability of any given host organisms to discriminate between self or non-self and activate defense responses against attempted microbial or pest/parasite infection. Plants utilize a multilayered immune system to protect themselves from invading pathogens or pests. One of the first layers of plant active defense is the ability of the host to sense microbes by perceiving microbe-associated molecular patterns (MAMPs). This type of recognition is mediated by pattern recognition receptors (PRRs) present at the cell surface and triggers a resistance response known pattern-triggered immunity (PTI) [1]–[3]. MAMP perception elicits a variety of defense responses including phosphorylation and dephosphorylation of proteins, production of reactive oxygen species (ROS), callose deposition and defense gene expression [4], [5]. Microbial pathogens evolved effectors to suppress PTI. In return, plant evolved resistance (R) proteins that recognize effectors direct or indirect and activate effector-triggered immunity (ETI) [1]. Frequently, ETI responses are dependent on the defense hormone salicylic acid (SA).

Root-knot nematodes (RKN; *Meloidogyne* spp.) are sedentary endoparasites of great agricultural importance. RKN are obligate biotrophs, penetrate the host roots behind the root cap and move towards the vascular cylinder where they initiate feeding on the cytoplasm of live cells and develop an elaborate feeding site known as giant cells. Cells around the feeding site undergo hyperplasia and hypertrophy resulting in the formation of galls, root symptoms associated with this group of nematodes [6]. Nematode salivary secretions have been implicated in development and maintenance of the feeding site [7]. Once feeding is initiated, RKN become sedentary and mature females lay eggs in gelatinous sacs protruding on the root surface. Although no information exists about how nematodes induce PTI, host defense responses against RKN are similar to biotrophic microbial pathogens and resistance to this pest is mediated by classical *R* gene responses frequently associated with cell death [8], [9].

Receptor like kinases (RLKs) are among the well characterized PRRs. Common features of the RLKs are the presence of an N-terminal signal sequence, an extracellular domain that varies in structure, a single membrane-spanning region, and a cytoplasmic protein kinase catalytic domain. RLKs with leucine-rich repeat (LRR)-containing extracellular domains comprise the largest subfamily of transmembrane RLKs in plants with over 200 members in *Arabidopsis thaliana* (Arabidopsis) [10], [11].

The LRR-RLK FLS2 (FLAGELLIN SENSING 2 (FLS2), belonging to LRR-RLK subfamily XII, was first identified in Arabidopsis by its ability to perceive the bacterial flagellin including the minimal epitope flg22 [12]. Responsiveness to flg22 is shared by members of all major clades of higher plants indicating that the PRR for this bacterial epitope is evolutionarily ancient and critical for antibacterial immunity. Interestingly, Arabidopsis *fls2* mutant plants, compromised in flg22 perception, are more susceptible to the bacterial pathogen *Pseudomonas syringae* pv. *tomato* (*Pst*) DC3000 only when spray inoculated and not when syringe infiltrated [13]. In contrast, *Fls2*-silenced *Nicotiana benthamiana* plants were more susceptible to both virulent and nonpathogenic *Pst* strains when syringe infiltrated [14], [15]. Besides *N. benthamiana*, orthologs of *FLS2* have been identified in several plant species including tomato (*Solanum lycopersicum*) [16].

In Arabidopsis, the *SOMATIC EMBRYOGENESIS RECEPTOR KINASE* (*SERK*) family consists of five LRR-RLKs belonging to subfamily II that share the presence of five LRRs in their extracellular domain [17]. These *SERK* family members play diverse roles in male sporogenesis, brassinosteroid (BR) response, PTI and cell death control [18]. The best-studied member of this family is *AtSERK3*. This kinase was independently identified as the BRASSINOSTEROID INSENSITIVE1 (BRI1)-ASSOCIATED KINASE1 (BAK1) in a genetic screen for suppressors of a weak *bri1* phenotype [19] as well as a BRI1 interacting protein in a yeast two-hybrid screen [20]. In addition, BAK1 directly interacts with BRI1 *in vivo* and the BAK1-BRI1 hetero-oligomers initiate BR-induced downstream signaling [21]. *bak1* null mutant plants display reduced sensitivity to BRs and reduced root growth inhibition by BR compared to wild type plants [19], [22]. Additional members of the family, *AtSERK1*, *AtSERK2* and *AtSERK4/BKK1* (*BAK1-like 1*), have also been implicated in BR signaling in a partially redundant role with *BAK1* [18], [22], [23]. *BAK1* also controls innate immunity independent from its function in BR signaling [24]–[28]. In combination with *BKK1*, *BAK1* regulates a cell-death signaling pathway as *bak1 bkk1* null double mutants display a dwarf phenotype, spontaneous cell death and seedling lethality [29]. In addition, both *BAK1* and *BKK1* contribute to basal disease resistance to the hemibiotrophic pathogen *Pst* and the biotrophic oomycete pathogen *Hyaloperonospora arabidopsidis* [28].

BAK1 forms flg22-induced complexes with FLS2, directly interacts with FLS2 and recognizes the C-terminus of the FLS2-bound flg22 [30]. *bak1* null mutants exhibit reduced flg22-responses including production of ROS, activation of mitogen-activated protein kinases (MAPK) and induction of defense genes indicating a role for this kinase in *FLS2*-mediated PTI [24], [25], [28]. BAK1 also forms complexes with additional PRRs and is required for responses triggered by a number of MAMPs from bacteria, fungi and oomycetes as well as signals generated from abiotic stresses such as cold shock and damage-associated molecular patterns indicating its role as a master regulator of stress responses [31].

In Solanaceous plants, *SERK3* homologs have been characterized from *N. benthamiana*, *N. attenuata* and tomato. In *N. benthamiana*, two *AtSERK3*/*BAK1* homologs, *NbSERK3A* and *NbSERK3B* were identified [32] while a single homolog *NaBAK1* has been reported from *N. attenuata* [33]. The entire tomato SERK family members have been identified [34], [35]. However, unlike Arabidopsis, tomato has only three *SERK*s (*Sl*SERK) members. These were named based on their phylogenetic relationship to the Arabidopsis SERKs as *SlSERK1*, *SlSERK3A* and *SlSERK3B* [34]. Interestingly, *SlSERK1* is required for potato aphid (*Macrosiphum euphorbiae*) resistance mediated by the presumed cytoplasmically localized nucleotide-binding (NB)-LRR R protein Mi-1, indicating a role for LRR-RLK in NB-LRR-mediated ETI [34]. Surprisingly, *SlSERK1* is not required for *Mi-1*-mediated resistance to RKN suggesting distinct recognition processes or signaling responses for aphids and nematodes.

Here, we describe the functional characterization of the remaining two *SlSERK*s, *SlSERK3A* and *SlSERK3B*, and their role in PTI to a bacterial pathogen and RKN. Using virus-induced gene silencing targeting *SlSERK3A* and *SlSERK3B* individually or combined revealed overlapping and unique roles for these *SlSERK3* paralogs in plant defense, cell death control and BR response. In addition, we show that both *Sl*SERK3A and *Sl*SERK3B co-immunoprecipitate with *Sl*FLS2 and partially complement the *bak1-4* null mutant.

## Results

### Molecular structure of *SlSERK3A* and *SlSERK3B*

The protein coding sequence (CDS) of *SlSERK3A* (1,848 bp) and *SlSERK3B* (1,854 bp) and their chromosome localization (chromosome 10 and 1, respectively) have been reported earlier [34]. The genomic sequences of both *SlSERK3A* and *SlSERK3B* were obtained from tomato cv. Moneymaker by amplifying overlapping regions based on cDNA sequences. Sequence analysis indicated that *SlSERK3A* genomic (KC261564) sequence is 10,874 bp in length while the *Sl*SERK3B genomic (KC261565) sequences is 7,965 bp. As predicted, *SlSERK3*A and *SlSERK3B* contain 11 exons (Figure S1). The predicted proteins of *Sl*SERK3A (616 amino acids, 68.28 kD) and *Sl*SERK3B (618 amino acids, 68.27 kD) have domains characteristic of SERK proteins including a signal peptide (with a putative cleavage site between amino acids 24 and 25 for *Sl*SERK3A or amino acids 29 and 30 for *Sl*SERK3B), a LRR N-terminal domain followed by four successive LRR domains, a Pro-rich region including a SPP motif, a single membrane-spanning domain and 11 conserved subdomains of a putative Ser/Thr protein kinase, followed by a short C-terminal (CT) tail [35] (Figure S2). Similar to BAK1, BKK1 and *At*SERK5, both *Sl*SERK3A and *Sl*SERK3B lack the LRR-CT domain present in *At*SERK1 and *At*SERK2. The levels of protein sequence identity of *Sl*SERK3A with *N. benthamiana* SERK3s and BAK1 and BKK1 proteins are: *Nb*SERK3A (96%), *Nb*SERK3B (96%), *At*SERK3/BAK1 (84%) and *At*SERK4/BKK1 (78%); while those of *Sl*SERK3B are: *Nb*SERK3A (91%), *Nb*SERK3B (89%), *At*SERK3/BAK1 (85%) and *At*SERK4/BKK1 (77%) (Figure S2).

### *Sl*SERK3A and *Sl*SERK3B are localized at the plasma membrane (PM)

**Figure 1:**
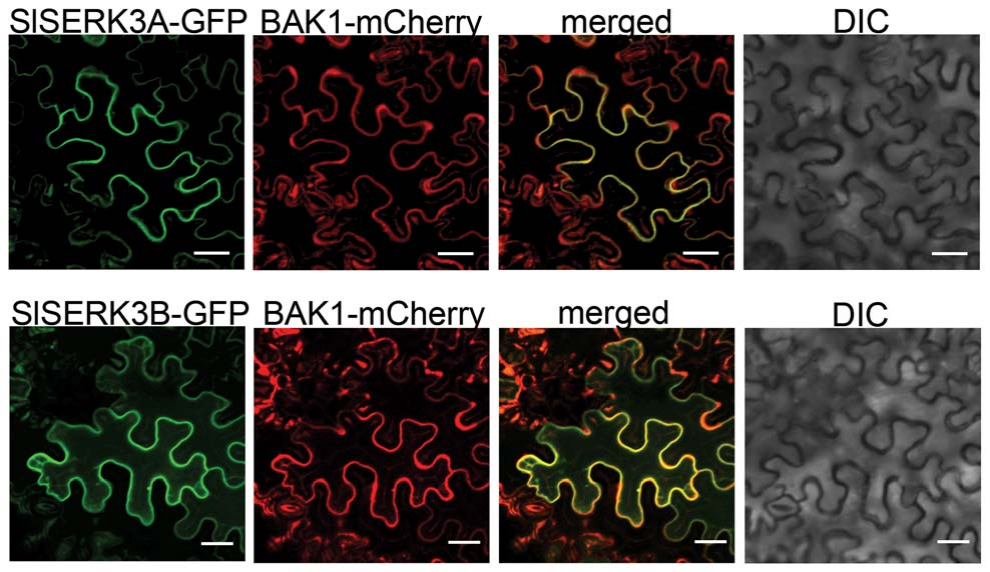
*Sl*SERK3A and *Sl*SERK3B co-localize with BAK1 at the plasma membrane (PM). *Agrobacterium*-mediated transient expression of *Sl*SERK3A-GFP or *Sl*SERK3A-GFP with BAK1-mCherry in *Nicotiana benthamiana* leaves. Localization of PM-associated BAK1-mCherry was compared with that of *Sl*SERK3A and *Sl*SERK3B (merged). Differential interference contrast (DIC) image. Leaf epidermal cells were imaged by confocal microscopy 72 h after infiltration with *Agrobacterium*. Bar = 20 µm.

Analysis of *Sl*SERK3A and *Sl*SERK3B protein sequences and their hydrophobicity profiles predicted a single transmembrane (TM) helix between the receptor-like part and the kinase domain, suggesting that *Sl*SERK3A and *Sl*SERK3B are TM proteins that are likely anchored to the PM, analogous to other SERK proteins [19], [34]–[37]. The subcellular localization of *Sl*SERK3A and *Sl*SERK3B was determined *in vivo* using translational fusions to green fluorescent protein (GFP) expressed by the p35S-*SlSERK3A*-GFP and p35S-*SlSERK3B*-GFP constructs. Confocal microscopy of *N. benthamiana* leaves transiently expressing these constructs in combination with the p35S-*BAK1*-mCherry construct, revealed that *Sl*SERK3A-GFP and *Sl*SERK3B-GFP are localized at a similar location as BAK1-mCherry mainly at the PM (Figure 1).

### *Sl*SERK3A and *Sl*SERK3B are active protein kinases

The presence of an Arg-Asp (RD) motif at the catalytic site in kinase subdomain VI and the conserved DFG motif in the kinase subdomain VII indicate that the LRR-containing *Sl*SERK3A and *Sl*SERK3B belong to the RD kinase LRR type-II subfamily of plant RLKs [38]. Comparison of the individual kinase subdomains of *Sl*SERK3A and *Sl*SERK3B with *At*SERKs revealed that the critical catalytic loop, which comprises a short stretch of residues in the kinase subdomain VI, is conserved. Recently, it has been shown that the cytoplasmic domains (CD) of *Sl*SERK3B to have kinase activity with the ability to autophosphorylate and transphosphorylate kinase inactive *Sl*BRI1-CD [39]. To test whether *Sl*SERK3A is also an active kinase, the CD of *Sl*SERK3A (residues 263 to 615), including the juxtamembrane, kinase domain and C-terminal parts, was produced in a heterologous system as GST-fusion proteins (GST-*Sl*SERK3A). As a control, the CD of *Sl*SERK3B (residues 259 to 616) was also produced as a GST fusion protein (GST-*Sl*SERK3B). We also developed the respective kinase-dead mutant variants *Sl*SERK3A* CD (D418N) and *Sl*SERK3B* CD (D420N), by introducing point mutations in the kinase catalytic loop based on a BAK1 kinase dead mutant [40], as GST- fusion proteins.

**Figure 2:**
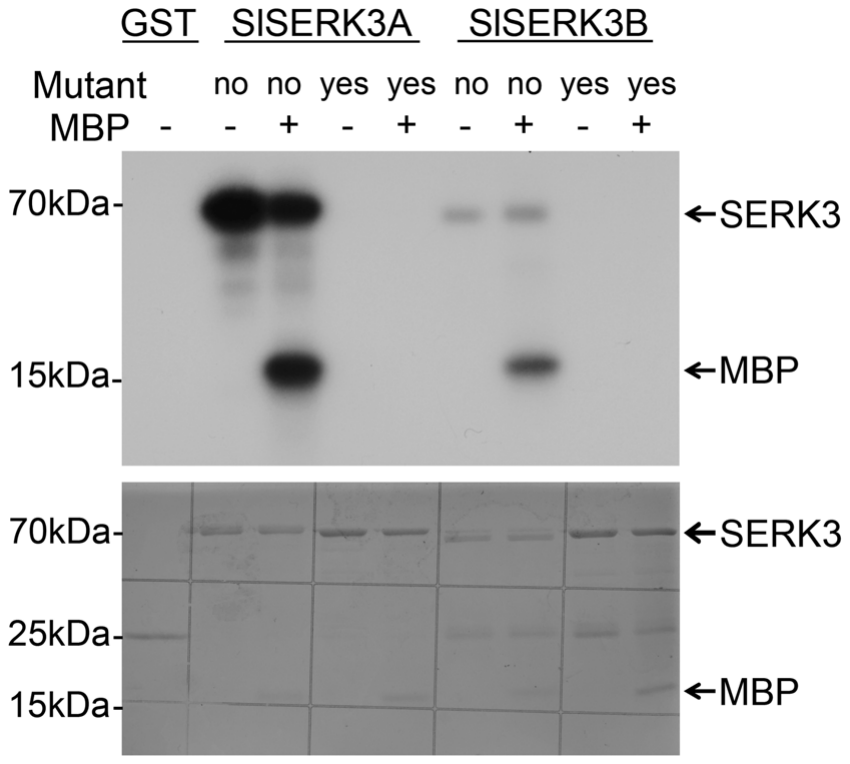
*Sl*SERK3A and *Sl*SERK3B are active protein kinases. Auto-phosphorylation and *trans*-phosphorylation of MBP were tested *in vitro* using freshly expressed and purified GST-tagged fusion proteins corresponding to the cytoplasmic domain of both *Sl*SERK3A and *Sl*SERK3B and their respective kinase dead mutants, *Sl*SERK3A* CD (D418N) and *Sl*SERK3B* CD (D420N). Proteins were fractionated on 12% SDS-PAGE. Coomassie blue stained and dried gel, lower panel; radiolabeled bands were revealed by autoradiography, upper panel. This experiment was repeated twice.

Purified proteins were subjected *in vitro* to an auto-phosphorylation assay as well as a trans-phosphorylation assay using the artificial substrate myelin basic protein (MBP). Analysis of the GST fusion proteins by SDS PAGE showed that both CD domains (66.32 and 67.64 kD) were soluble, and migrated as single bands at their predicted molecular masses (Figure 2, lower panel). A band corresponding to each of the auto-phosphorylated *Sl*SERK3A and *Sl*SERK3B proteins was observed when the kinase domains were used alone or in combination with MBP (Figure 2). In the presence of the wild type kinase domains, a phosphorylated MBP band was observed. As expected, both auto-phosphorylation and trans-phosphorylation of MPB were abolished by the kinase dead mutants of each *Sl*SERK3* CD (Figure 2). Although kinase activity, both auto-phosphorylation and trans-phosphorylation, is stronger for *Sl*SERK3A CD compared to *Sl*SERK3B CD (Figure 2), this pattern of kinase activity was not consistently observed in replicated experiments. Taken together these results indicate that similar to *Sl*SERK3B, *Sl*SERK3A is also an active kinase catalyzing *in vitro* both auto- and trans-phosphorylation.

### *SlSERK3A* and *SlSERK3B* control cell death

**Figure 3:**
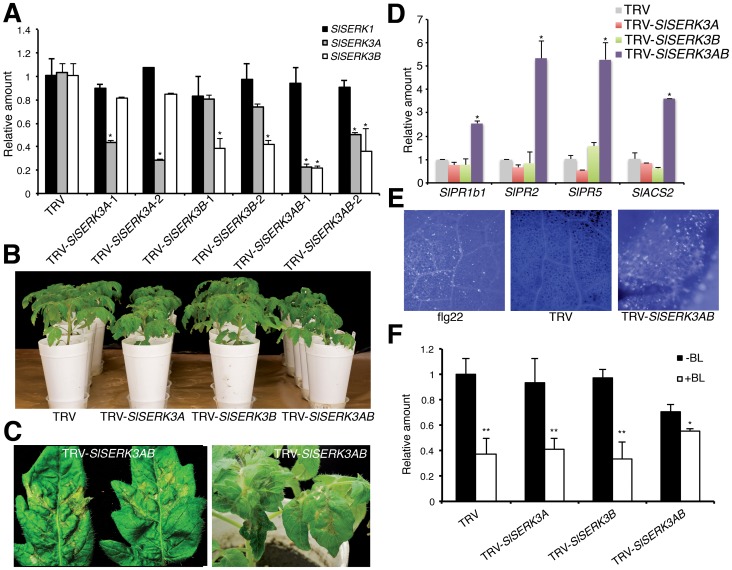
*SlSERK3A* and *SlSERK3B* co-silenced plants are compromised in cell death control and BR sensitivity. (**A**) Transcript levels of VIGS-silenced genes were evaluated using qRT-PCR. Tomato cv. Moneymaker plants treated with TRV empty vector (TRV), TRV-*SlSERK3A*, TRV-*SlSERK3B* or TRV-*SlSERK3AB* were evaluated. Expression was normalized against *UBI3*. Two independent samples were analyzed per construct. Values are average ± SE of three technical replicates. *P<0.05 significant difference from TRV (two-sample *t*-test). Experiment was repeated three times with similar results. (**B**) Phenotype of individually silenced *SlSERK3A*, *SlSERK3B* and co-silenced plants. (**C**) Cell death lesions in *SlSERK3A* and *SlSERK3B* co-silenced tomato leaflets. (**D**) Defense and senescence-related *SlPRIa*, *SlPR2*, *SlPR5*, and *SlACS2* gene regulation in *SlSERK3A or SlSERK3B* silenced and co-silenced plants. Transcript levels were evaluated using qRT-PCR normalized against *SlUBI3*. Values are average ± SE (n = 3). * indicates significance difference from TRV at *P*<0.05 (two-sample t-test). Experiment was repeated twice with similar results. (**E**) Aniline blue-stained tomato leaf discs. Callose accumulation was detected near the edges of leaf patches showing cell-death in co-silenced *SlSERK3A* and *SlSERK3B* plants and TRV control. Leaves treated with 1 µM flg22 for 24 h were used as control. (**F**) Leaflets of tomato plants silenced for *SlSERK3A*, *SlSERK3B* or co-silenced and TRV control were treated with 10 µM BL for 12 h for *SlCPD* expression evaluation. Transcript levels were evaluated using qRT-PCR normalized against *SlUBI3*. Values are average ± SE (n = 3). *P<0.05 and **P<0.01 indicate significant difference from the respective –BL control (two-sample *t*-test). This experiment was repeated twice with similar results.

To assess the functional roles of *SlSERK3A* and *SlSERK3B*, we developed gene-specific silencing constructs able to suppress *SlSERK3A* or *SlSERK3B* transcripts using virus-induced gene silencing (VIGS). A third construct was developed to co-silence both *SlSERK3A* and *SlSERK3B* (Figure S3). The target specificities of the VIGS constructs in tomato were confirmed using quantitative RT-PCR (Figure 3A and Figure S4A). Co-silencing both *SlSERK3A* and *SlSERK3B* in tomato cultivar Moneymaker resulted in plants exhibiting reduced growth (Figure 3B) and spontaneous cell death in leaves (Figure 3C). Silencing *SlSERK3A* also reduced plant growth albeit to a lesser degree than the co-silenced plants, while silencing *SlSERK3B* did not have any obvious effect on plant growth (Figure 3B). Silencing either *SlSERK3A* or *SlSERK3B* individually did not result in spontaneous cell death (Figure S4B).

To investigate the molecular mechanism leading to the cell death phenotype in the co-silenced plants, we examined expression of the defense and senescence-related genes *SlPR1b1*, *SlPR2*, *SlPR5*, and *SlACS2* ([41]; Table S1). Expression of all four genes was upregulated in *SlSERK3A SlSERK3B* co-silenced leaves (Figure 3D). This overall expression is similar to transcript patterns for the respective Arabidopsis orthologs reported for *bak1-4 bkk1-1* double mutant [29]. Strikingly, expression of none of these four genes was upregulated in plants individually silenced for *SlSERK3A* or *SlSERK3B* (Figure 3D). Tomato leaflets individually silenced for *SlSERK3A* or *SlSERK3B* or co-silenced, were further evaluated for callose deposition a known cell death-associated defense response. Aniline blue staining of individually silenced leaflets did not reveal callose deposition (Figure S4C), while callose deposits were detected in co-silenced leaflets in areas near tissues exhibiting cell death (Figure 3E). Co-silenced leaflets were also evaluated for cell-death associated H_2_O_2_ accumulation. In similar regions near dead tissues, H_2_O_2_ accumulation was detected as brown spots using 3,3′-diamino benzidine (DAB) staining (Figure S5A). Taken together, these results indicate that *SlSERK3A* and *SlSERK3B* have a redundant function in suppressing cell death. Because of the spontaneous nature of the cell death phenotype, co-silenced plants were not included in defense related experiments.

*SlSERK3A* and *SlSERK3B* co-silenced plants were smaller in overall stature compared to TRV-empty vector (TRV) control plants (Figure 3B). The semi-dwarf stature suggested BR-deficiency or -response in these plants. In Arabidopsis, expression of the *CPD* gene, involved in BR biosynthesis, is downregulated by BR treatment and this downregulation is compromised in the *bri1* mutant as well as in most double and triple mutants of *bak1* with other *Atserks* but not in any single *Atserk* mutant [22], [42]. To assess whether *SlSERK3* silenced plants were affected in BR response, *SlCPD* gene expression was evaluated in plants treated or untreated with BR. Basal *SlCPD* transcript levels were similar in TRV control plants and plants individually silenced for *SlSERK3A* or *SlSERK3B* (Figure 3F). However, a reduction in *SlCPD* transcript levels was observed in *SlSERK3A* and *SlSERK3B* co-silenced plants suggesting that *SlSERK3A* or *SlSERK3B* are required for the basal expression of *SlCPD* (Figure 3F). Similar to Arabidopsis, exogenous application of BR downregulated *SlCPD* transcript levels in tomato TRV control plants (Figure 3F). In addition, downregulation of *SlCPD* transcript levels in response to BR was not affected in tomato plants individually silenced for *SlSERK3A* or *SlSERK3B* (Figure 3F and Figure S5B). In contrast, downregulation of *SlCPD* transcript levels was greatly compromised in the *SlSERK3A* and *SlSERK3B* co-silenced plants treated with BR (Figure 3F and Figure S5B) suggesting a redundant function for these two paralogs in BR signaling.

### *SlSERK3A* and *SlSERK3B* are required for disease resistance

**Figure 4:**
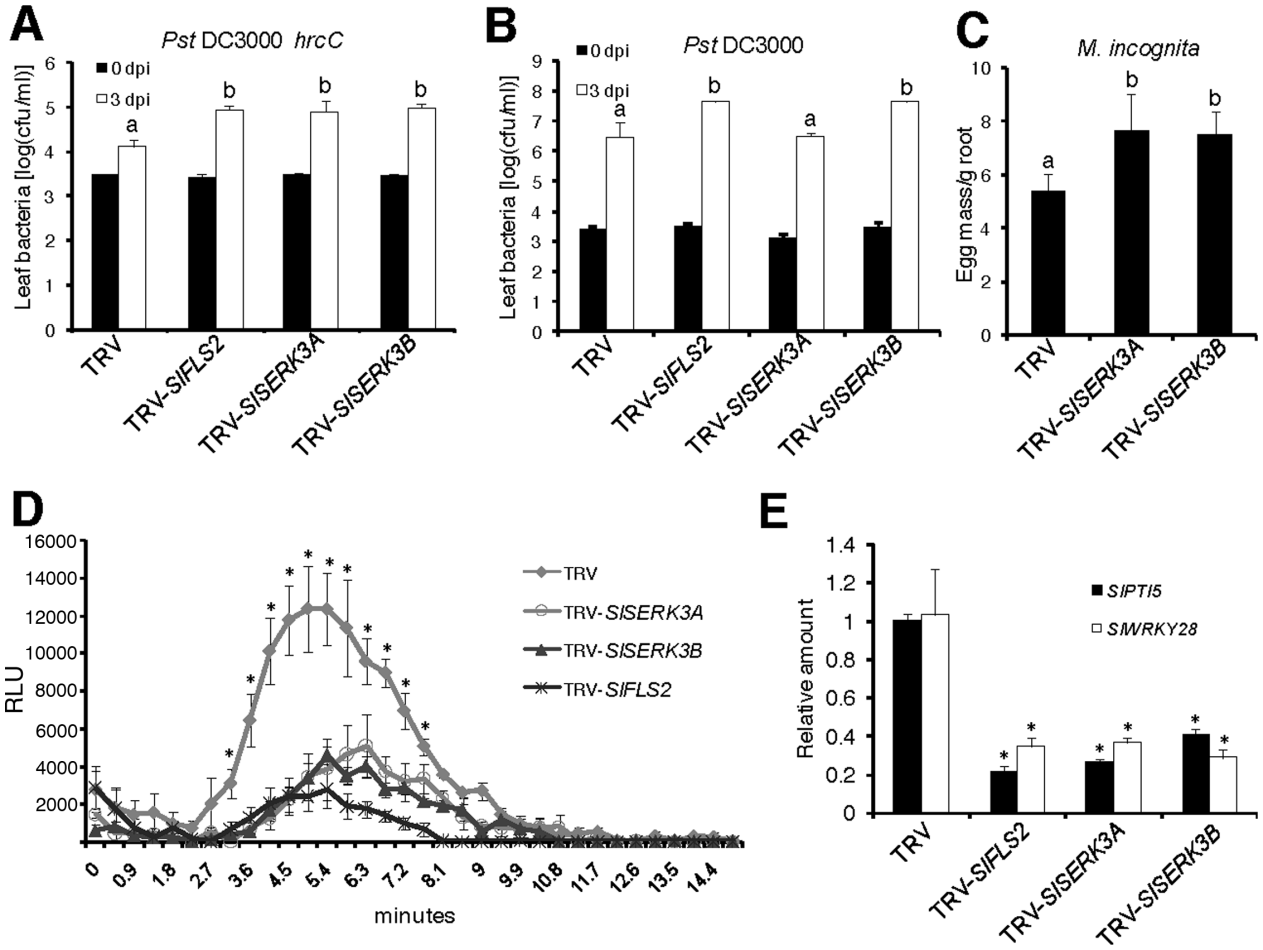
*SlSERK3A and SlSERK3B* are involved in PTI in tomato. Five-week-old tomato plants cv. Moneymaker silenced for *SlFLS2*, *SlSERK3A* or *SlSERK3B* and TRV control were used. (**A**) and (**B**) Plants were vacuum infiltrated with *Pst* DC3000 *hrcC* or *Pst* DC3000 and bacterial counts were performed at 0 and 3 days post infiltration (dpi). Results are average (±) SE (n = 5). Letters above the graphs denote significance difference at *P*<0.01 (ANOVA Tukey HSD test). These experiments were repeated twice with similar results. (**C**) Plants were infected with 1,000 J2 each and evaluated 6 weeks later. No *FLS2* silenced plants were used in this assay. Results are average (±) SE (n = 9). Letters above the graphs denote significance difference at *P*<0.05 (ANOVA Tukey HSD test). This experiment was repeated once with similar results. (**D**) Leaf samples were floated on water overnight. ROS burst was measured as relative light units (RLUs) emitted in a luminol-based assay within 15 min after 1 µM flg22 treatment. Values are average ± SE (n = 4). * indicates statistically significant difference from TRV at *P*<0.05 (two-sample *t*-test). This experiment was repeated once. (**E**) TRV-treated plants were vacuum infiltrated with *Pst* DC3000 *hrcC* and harvested 6 h later. Expression was evaluated using qRT-PCR normalized against *SlUBI3*. Values are average ± SE (n = 3). *P<0.05 and ** *P*<0.001 significant difference from TRV (two-sample *t*-test). This experiment was repeated twice with similar results.

To investigate a possible role for a single *SlSERK3* gene in disease resistance, we evaluated *SlSERK3A* or *SlSERK3B* silenced plants for resistance to the tomato pathogen *Pst* DC3000 and its nonpathogenic *hrcC* mutant derivative *Pst* DC3000 *hrcC*. To develop a control, we targeted the tomato flagellin receptor *SlFLS2* (Figure S3) [16] for silencing in tomato and vacuum infiltrated the silenced plants (Figure S6) with *Pst* DC3000 *hrcC* and *Pst* DC3000. Silencing *SlFLS2* enhanced the growth of both *Pst* DC3000 *hrcC* and *Pst* DC3000 relative to TRV control plants (Figure 4A and 4B). Importantly, silencing either *SlSERK3A* or *SlSERK3B* (Figure S4A) also enhanced growth of *Pst* DC3000 *hrcC* indicating non-redundant roles for *SlSERK3*s in PTI against non-pathogenic *Pst* (Figure 4A). Interestingly, silencing *SlSERK3B* and not *SlSERK3A* resulted in enhanced *Pst* DC3000 growth suggesting an additional role for *SlSERK3B* in bacterial defense that may be distinct from its role in PTI against the non-pathogenic *Pst* strain (Figure 4B).

Root-knot nematodes are serious tomato pests and no information exists on PTI for this group of pests. We wondered whether resistance to nematodes might also involve PTI and the likely requirement for the master PTI regulator *SERK3*. To address this, we infected tomato plants silenced for *SlSERK3A* or *SlSERK3B* with *Meloidogyne incognita* infective-stage juveniles and evaluated the roots for nematode infection and reproduction. Root weights of tomato plants silenced for either *SlSERK3A* or *SlSERK3B* (Figure S7A) were similar to TRV control plants (Figure S7B). Interestingly, plants silenced for either *SlSERK3A* or *SlSERK3B* exhibited enhanced susceptibility to RKN compared to TRV control indicating a likely role for PTI in RKN resistance (Figure 4C). As reported earlier [43], VIGS in tomato roots was patchy (Figure S7A) suggesting that the enhanced susceptibility values reported in this assay are likely an underestimate.

### *SlSERK3A* and *SlSERK3B* are required for flg22-triggered immunity

To further characterize the role of *SlSERK3*s in bacterial defense, we evaluated ROS production in *SlSERK3A-*, *SlSERK3B-* or *SlFLS2-*silenced plants. Tomato silenced for *SlSERK3A* or *SlSERK3B* were severely reduced in flg22-triggered ROS production, similar to *SlFLS2* silenced plants, consistent with their role as positive regulators of bacterial PTI (Figure 4D). To confirm attenuation of PTI in *SlSERK3*-silenced plants, expression of known PTI marker genes [15], [44] was investigated. Transcripts of both *SlPTI5* and *SlWRKY28* were upregulated within 6 h after *Pst* DC3000 *hrcC* treatment in TRV-treated leaves (Figure 4E). In contrast, silencing *SlFLS2*, *SlSERK3A* or *SlSERK3B* severely reduced this up-regulation of both genes (Figure 4E). The observed attenuation of ROS production and reduction in defense marker gene induction further confirmed the role of *SlSERK3*s in tomato PTI.

### *Sl*SERK3A and *Sl*SERK3B form a flg22-induced complex with *Sl*FLS2 in *N. benthamiana*

**Figure 5:**
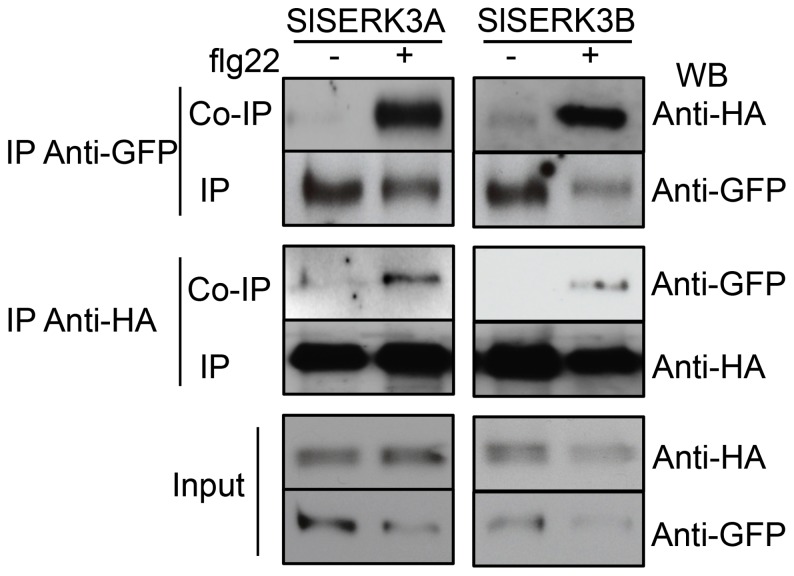
*Sl*FLS2 co-immunoprecipitates with *Sl*SERK3A and *Sl*SERK3B. *Nicotiana benthamiana* leaves transiently expressing *Sl*SERK3A-HA or *Sl*SERK3B-HA constructs and *Sl*FLS2-GFP were elicited (+) or not (−) with 100 nM flg22 for 5 min. Total proteins (input) were subjected to reciprocal immunoprecipitation and immunoblotting. Immunoprecipitation with anti-GFP Protein A agarose beads (upper panel) or anti-HA (middle panel). This experiment was repeated once with similar results. WB: Western blot.

To test whether *Sl*FLS2 heterodimerizes with *Sl*SERK3A or *Sl*SERK3B *in vivo*, we transiently co-expressed a *Sl*FLS2-GFP fusion protein with either *Sl*SERK3A-HA or *Sl*SERK3B-HA fusion proteins in *N. benthamiana* for co-immunoprecipitation (Co-IP) experiments. Within 5 minutes after flg22-treatment, interactions between *Sl*FLS2 and either *Sl*SERK3A or *Sl*SERK3B were detected by Co-IP with anti-GFP and immunoblotting with anti-HA, (Figure 5). Neither *Sl*SERK3A or *Sl*SERK3B were detected in the untreated anti-GFP immunoprecipitates (Figure 5). Reciprocal Co-IP using anti-HA to precipitate *Sl*SERK3A or *Sl*SERK3B and immunoblotting with anti-GFP, detected *Sl*FLS2 only in flg22-treated samples (Figure 5). These results suggest flg22-induced complex formation between *Sl*FLS2 and *Sl*SERK3A or *Sl*SERK3B consistent with the ligand dependency of the *At*FLS2-BAK1 association [24], [25], [45], [46].

### Heterologous expression of *SlSERK3A* and *SlSERK3B*

**Figure 6:**
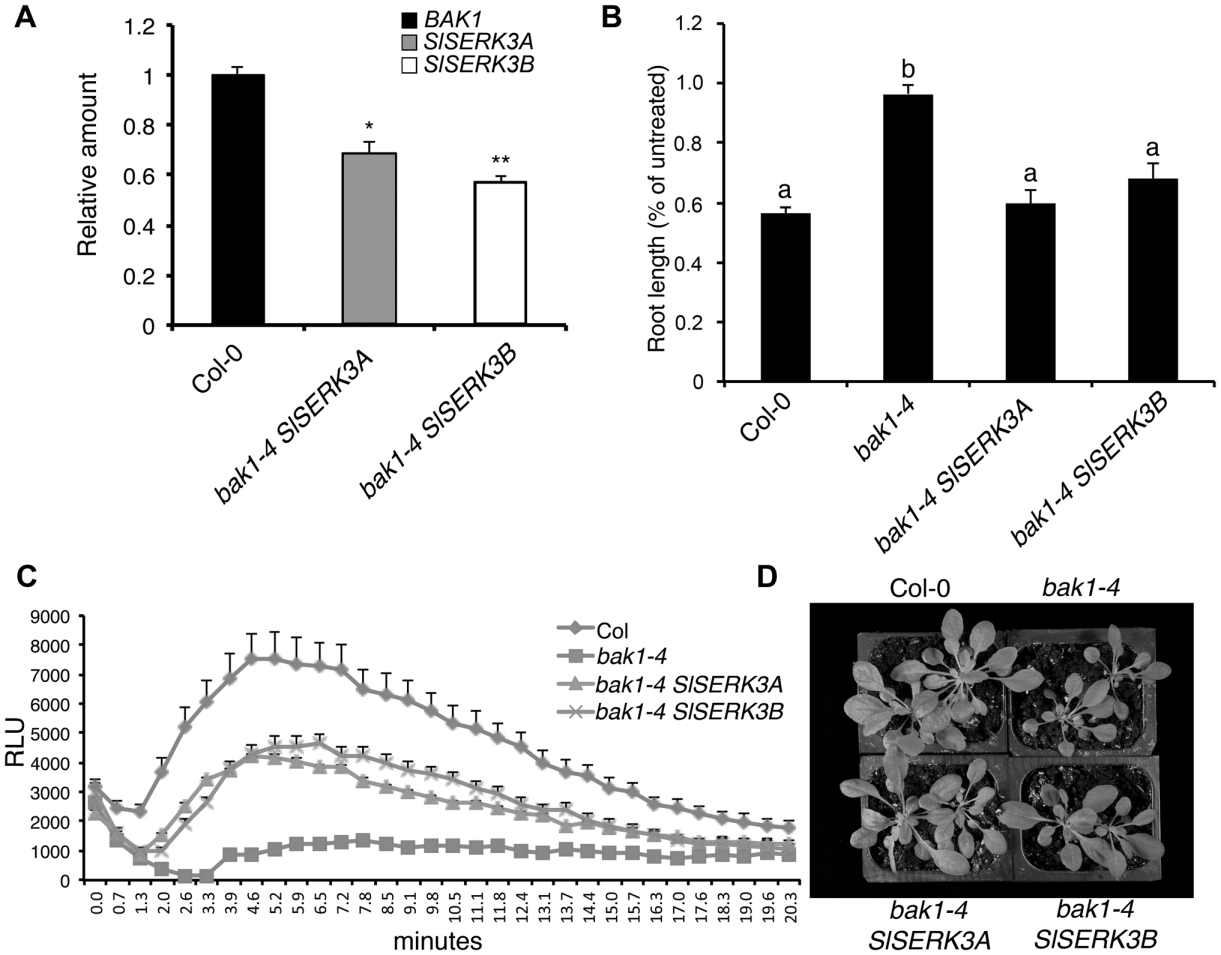
*SlSERK3A* and *SlSERK3B* partially complemented the Arabidopsis *bak1-4* null mutant. (**A**) *SlSERK3A and SlSERK3B* transcript levels in transgenic *bak1-4* plants expressing pBAK1-*SlSERK3A (bak1-4 SlSERK3A)* or pBAK1-*SlSERK3B (bak1-4 SlSERK3B)* were evaluated using qRT-PCR. Values are average ± SE (n = 3) normalized relative to *AtActin* and calibrated to expression of *BAK1* in Col-0. *P<0.05 and ** *P*<0.001 significant difference from Col-0 (two-sample *t*-test). (**B**) Relative root growth of 9-day-old seedlings grown on medium with or without 1 nM BL. Root length is presented relative to untreated control for each genotype. Values are average ± SE (n = 50). Values were arcsine transformed for statistical analysis. Letters above the graphs denote significance difference at *P*<0.01 (ANOVA Tukey HSD test). This experiment was repeated twice with similar results. (**C**) Leaf discs were floated on water overnight. ROS burst was measured using a luminol-based assay within 25 min after 1 µM flg22 treatment. Values are average ± SE (n = 8). (**D**) A photo of representative short-day grown 4.5-week-old Arabidopsis plants of the indicated genotypes.

To determine whether *Sl*SERK3A or *Sl*SERK3B are the functional orthologs of BAK1, we performed complementation tests with the *A. thaliana bak1-4* mutant. We introduced *Sl*SERK3A or *Sl*SERK3B expression constructs containing the Arabidopsis *BAK1* promoter, into the *bak1-4* null mutant background and developed stable transgenic plants. The *bak1-4* mutant has reduced sensitivity to exogenous BR treatments and displays semi-dwarf phenotype when grown under short-day conditions [24]. Root growth assays showed that transgenic *bak1-4* mutant plants expressing *SlSERK3A* or *SlSERK3B* (Figure 6A) exhibited restored wild-type sensitivity to exogenous BR treatment (Figure 6B and Figure S8). These complementation lines also exhibited an intermediate growth phenotype compared to wild type Col-0 and the *bak1-4* mutant (Figure 6D). In addition, the complemented plants showed enhanced flg22-induced ROS production compared to the *bak1-4* mutant albeit ROS levels were lower than in wild type Col-0 (Figure 6C).

## Discussion

In Solanaceae, SERK members have been identified in tomato, *N. benthamiana* and *N. attenuata*. However, it is not clear how many SERKs members *Nicotiana* species have. Only for tomato, all members of this family have been identified and unlike Arabidopsis, that has five members, tomato was found to have only three members [34]. In both tomato and *N. benthamiana*, two members have particularly high levels of sequence similarity to *AtSERK3*/*BAK1* suggesting recent duplication events in the lineage of these solanaceous species. Although a role for *NbSERK3* has been identified in microbial pathogen defense [25], [32], it is not clear which of the two *NbSERK3* paralogs contribute to the resistance and whether the two members have redundant roles in defense. Similarly, *SlSERK3* is required for the resistance to the vascular fungal pathogen *Verticillum* mediated by the receptor like protein (RLP) Ve1 and for defense responses induced by the fungal *Ethylene-induced xylanase (Eix)* mediated by RLP LeEix [47], [48]. In these tomato and *N. benthamiana* studies, VIGS was used to evaluate the defense related roles of *SERK3* and because of the high level of sequence identity between the two *SERK3* paralogs from each plant species, the VIGS constructs used are capable to silence both members. However, the specificity of silencing was not evaluated in these experiments, consequently, the specific function of the individual paralog remains unclear. In this work, we were able to specifically silence individual *SlSERK3A* and *SlSERK3B* and co-silenced them by designing VIGS constructs partially targeting the respective untranslated gene regions. This allowed us to dissect the contributions of each of these gene paralogs and identify common and distinct roles for them.

*SlSERK3A* silenced plants were smaller in size compared to TRV control or *SlSERK3B* silenced plants which could be due to pleiotropic effect on BR signaling. However, molecular data indicate that individually silenced *SlSERK3A* plants are not affected in BR signaling (Figure 3F). Although the reason for *SlSERK3A* silenced plant short stature is unclear, our data indicate that BR signaling is not affected at a detectable level in either *SlSERK3A* or *SlSERK3B* silenced plants.

Interestingly, vacuum infiltration of *FLS2*-silenced tomato plants with *Pst* resulted in significant increase in bacterial growth a similar phenotype seen in *FLS2*-silenced *N. benthamiana* plants [14], [15]. This is in contrast to Arabidopsis *fls2* mutant on which, compared to wild-type plants, no bacterial growth difference was observed after syringe infiltration [13]. Lower bacterial growth was seen on the *fls2* mutant only when bacteria were spray inoculated [13]. Our result with tomato, combined with that from *N. benthamiana*, indicates that flagellin perception by FLS2 in *Solanaceae* functions in the mesophyll cells while this perception in Arabidopsis is active in the guard cells. It remains to be determined whether FLS2 perception in *Solanaceae* functions also in the guard cells.

Our results showed that both *SlSERK3* paralogs contributed to resistance against the non-pathogenic *Pst* DC3000 *hrcC* strain and to RKN, while only *SlSERK3B* promoted resistance against virulent *Pst* DC3000. This indicates that these two *SERK3* members have evolved distinct immune related functions. The bacterial defense related role of *SlSERK3B* is similar to that of *BAK1*, as shown with the *bak1-5* mutant, suggesting that this tomato SERK member is an authentic *BAK1* ortholog [28]. However, no clear *SlSERK3A* Arabidopsis ortholog can be identified based only on its defense function in tomato. Although a role in bacterial defense has been demonstrated for *AtSERK4/BKK1*, the closest *BAK1* paralog, this role is only detectable in the *bak1-5 bkk1* double mutant infected by a weekly virulent coronatine defective *Pst* strain [28]. Thus, *BKK1* appears only to play a minor role in bacterial defense, unlike *SlSERK3A* which strongly contributes to basal resistance against *Pst* DC3000 *hrcC*. Both *BAK1* and *BKK1* have non-redundant basal resistance functions against fungal and oomycete pathogens [28]. Although *SERK3* paralogs have been implicated in fungal resistance in tomato it remains unclear which one of them contributes to this defense function because of the reasons stated above.

Recently the presence of PTI in roots was demonstrated using the well-known MAMPs chitin, flg22 and peptidoglycans and the immune responses to the latter two MAMPs were *BAK1*-dependent [49]. In addition, immunity function has been attributed to the *bak1-4* Arabidopsis mutant to *Verticillium* indicating a role for *BAK1* in basal defense to vascular pathogens [50]. Our results showing enhanced RKN susceptibility of *SlSERK3A* or *SlSERK3B* silenced plants indicate a role for *SERK3* in resistance to RKN and the likely existence of PTI by nematode-associated molecular pattern(s). Silencing *SlSERK3A* resulted in reduced plant size and may be affected in BR signaling. However, it is unlikely that the enhanced RKN susceptibility is due to altered BR signaling as *SlSERK3B* silenced plants also exhibited enhanced RKN susceptibility but did not have altered plant size phenotype or are affected in BR signaling.

A number of nematode parasitism genes have been reported that play roles in virulence and suppression of host defenses [9], [51], [52]. However, no nematode-derived molecular patterns have been yet identified and it is difficult to speculate as to the nature of this pattern. Proteinaceous salivary secretions originating from esophageal gland cells have been implicated in nematode root invasion and migration as well as initiation and maintenance of their elaborate feeding sites [7]. Other sources of secretions from the nematode could be from sensory structures such as amphids or phasmids, or the excretory pore or the cuticle, none of which have been implicated in interactions with their hosts. Moreover, nematode penetration, feeding and secretion of cell wall degrading enzymes potentially produce damage-associated molecular patterns which could be the source of the nematode induced PTI. New research is needed to investigate nematode-induced PTI and to identify the nature of the nematode-associated molecular pattern(s) and its cognate PRR.

Our results showed that *SlSERK3A* and *SlSERK3B* have a redundant function in suppressing cell death. A similar function has been attributed to *BAK1* and *BKK1* [29], indicating that these *SERK* paralogs share similar cell death control functions in both Arabidopsis and tomato. However, co-silencing *SERK3A* and *SERK3B* in *N. benthamiana* does not result in cell death indicating this redundant cell death suppression function for two *SERK* members is not universal among plant species [32]. In Arabidopsis, it is speculated that these two SERK members suppress cell death through their interaction with the RLK, BIR1 (BAK1-Interacting Receptor Like Kinase 1) that possibly perceives an endogenous survival signal(s) [53]. As an alternative, it is discussed that SERK-associated PTI signaling complexes are guarded by R proteins [53], [54]. In the latter case, the absence of both BAK1 and BKK1 may constitutively activate R protein-mediated defense responses, including cell death. Such a scenario is supported by the fact that the cell death phenotype in the *bak1 bkk1* double mutant is dependent on the defense hormone SA which is required for many R protein-dependent immune responses [29]. The constitutive activation of the SA-regulated gene, *SlPR1b1* [55], we observed in *SlSERK3A* and *SlSERK3B* co-silenced plants suggests that the cell death phenotype in these tomato plants is also SA-regulated and could be triggered by an R protein. It remains to be seen whether an R protein guards SERK3-associated PTI signaling complexes in tomato.

*Sl*SERK3A and *Sl*SERK3B belong to the RD class of Ser/Thr kinases that share a conserved catalytic core. All *At*SERK family members are active kinases and able to autophosphorylate *in vitro* [19], [56]. Similarly, both *Sl*SERK3A and *Sl*SERK3B are active kinases able to auto-phosphorylate and trans-phosphorylate MPB *in vitro*. Multiple Ser and Thr residues are auto-phosphorylated in *Sl*SERK3B-CD only a subset correspond to auto-phosphorylated Arabidopsis BAK1-CD residues [39]. It remains to be seen whether these additional conserved residues are also auto-phosphorylated in *Sl*SERK3A-CD.

As the single amino acid mutation that eliminated the kinase activity of BAK1 also eliminated the kinase activities of both *Sl*SERK3A and *Sl*SERK3B [40], our data showed that the catalytic kinase core between BAK1 and the two *Sl*SERK3 paralogs are structurally and functionally conserved. Although the substrates of most SERK members are not well defined, it is well documented that BAK1 trans-phosphorylates a number of RLKs [19], [20], [40], [45], [57]. Recently it has been shown that *Sl*SERK3B can trans-phosphorylate *Sl*BRI [39]. Based on the high sequence similarity between the *Sl*SERK3A and *Sl*SERK3B catalytic domains, and their redundant role in BR signaling, we hypothesis that *Sl*SERK3A can also trans-phosphorylate *Sl*BRI. Since *SlSERK3* is required for signaling and immunity mediated by the tomato RLP *LeEix2* and *Ve1*, respectively, it is likely that *Sl*SERK3A or *Sl*SERK3B individually or together are capable of trans-phosphorylating multiple receptors in a manner similar to BAK1.

Both *Sl*SERK3A and *Sl*SERK3B formed flg22-dependent complex with *Sl*FLS2. Several *At*SERK members are able to heterodimerize with FLS2, albeit at variable levels of association, in ligand dependent manner [25], [28]. Further investigations should reveal whether *Sl*SERK3A and *Sl*SERK3B form a heterodimer in this interaction and whether *Sl*FLS2 is also able to heterodimerize with *Sl*SERK1.

The ability of *Sl*SERK3A and *Sl*SERK3B to form a ligand induced complex with *Sl*FLS2 suggested a role for these two *SERK* paralogs in *FLS2*-dependent signaling. Indeed, both *SlSERK3A* and *SlSERK3B* have non-redundant functions in flg22-induced ROS production and activation of defense related genes. Among Arabidopsis *SERK* members, a similar function is only demonstrated for *BAK1*. Only *bak1* single mutants are compromised in flg22-induced ROS and none of the remaining individual *serk* null mutants are impaired in ligand induced ROS production [24], [28]. A minor role in flg22-induced ROS production was uncovered for *bkk1* in the *bak1-5 bkk1* double mutant [28]. Taken together, this information indicates that *SlSERK3A* and *SlSERK3B* have *BAK1*-related functions and seem to be true orthologs of this Arabidopsis gene. Indeed, in complementation experiments either *SlSERK3A* or *SlSERK3B* partially rescued the *bak1* mutant phenotype. The lack of full *bak1* complementation is likely due to sequence divergence between these Arabidopsis and tomato orthologs.

Similar to Arabidopsis, the expression of *SlCPD* in tomato is down-regulated by exogenous application of BR via a presumed negative feedback mechanism [42]. The attenuation of *SlCPD* responsiveness to exogenous BR application in the *SlSERK3A* and *SlSERK3B* co-silenced plants, and not in the individual *SlSERK3A* or *SlSERK3B* silenced plants, indicates that *SlSERK3A* and *SlSERK3B* have redundant function in BR signaling. Since the *SlSERK3A* and *SlSERK3B* co-silenced plants had residual BR signaling competence, it suggests that the only other family member *SlSERK1* also contributes to BR response. Based on *CPD* expression analysis in Arabidopsis, it is not clear the contribution of *BKK1/SERK4* to BR signaling as BR effect on *CPD* expression have been analyzed in the double mutant *bak1 serk1* or the triple mutant *bak1 bkk1 serk1* and not in the double mutant *bak1 bkk1* [22]. Nonetheless, our results show that *SlSERK3A* and *SlSERK3B* contribute to most of the BR effect on *CPD* expression in tomato.

In summary, our work provides functional characterization of *SlSERK3A* and *SlSERK3B* in an important crop and demonstrates differences and similarities in the role of *BAK1* and *SlSERK3A* and *SlSERK3B* paralogs in immunity and BR signaling. This work also provides a foundation for future characterization of PTI against RKN in roots.

## Materials and Methods

### Plant material and growth conditions

One-week-old tomato (*Solanum lycopersicum*) cv. Moneymaker seedlings were transplanted into California mix II or sand. Plants were maintained in plant growth rooms at 24°C before VIGS treatment and then at 19°C until use in bioassays with a 16 h light and 8 h dark photoperiod. *Nicotiana benthamiana* plants were maintained in a plant growth room at 24°C at a similar photoperiod. Plants were fertilized biweekly with MiracleGro (Stern's MiracleGro). *Arabidopsis thaliana* (Arabidopsis) Col-0 and T-DNA insertion null mutant *bak1-4* (SALK_116202) plants were grown in soil under fluorescent lights (10 h light and 14 h dark, 100 µEinstein/m^2^/s) at 22°C.

### Virus-induced gene silencing (VIGS)

The TRV-*SlSERK3A* (contains 152 bp of the *SlSERK3B* gene (+1837 to +1988)), TRV-*SlSERK3B* [contains 174 bp of the *SlSERK3B* gene (+1844 to +2017)], TRV-*SlSERK3AB* [contains 178 bp of the *SlSERK3B* gene (+1289 to +1466)] and TRV-*SlFLS2* [contains 111 bp of the *SlFLS2* gene (+3460 to +1570)] were constructed by amplifying the desired fragments using gene-specific primers (Table S2) and tomato cv. Moneymaker cDNA, and recombining into Gateway compatible pDONR207 vector (Invitrogen) and finally into pTRV2. After sequence verification, constructs were transformed into *A. tumefaciens* strain GV3101.

VIGS was performed using the bipartite TRV (pTRV1 and pTRV2; [58]) vector in *Agrobacterium tumefaciens* and syringe infiltration (agroinfiltration) of 2-week-old tomato leaflets. Equal volumes (OD_600_ = 1) of *A. tumefaciens* pTRV1 and suspensions containing pTRV2-derived constructs, pTRV2 empty vector or TRV-*PDS* were mixed before infiltration [58].

### Constructs

The coding sequences (CDS) of *SlSERK3A* and *SlSERK3B* were PCR amplified from tomato cDNA using the primers given in Table S2. The *BAK1* promoter was amplified from Arabidopsis genomic DNA using primers listed in Table S1 and fused with the CDS of either *SlSERK3A* or *SlSERK3B* and cloned into pDONR207 (pBAK1-*SlSERK3A* and pBAK1-*SlSERK3B*). The CDS without stop of *SlSERK3A*, *SlSERK3B* and *SlFLS2* were PCR amplified from tomato cDNA using primers listed in Table S1 and cloned into pDONR207. All resulting constructs were sequence verified.

pENTR207 (pBAK1-*SlSERK3A*) and pENTR207(pBAK1-*SlSERK3B*) were recombined into pEarleyGate303. pENTR207-*SlFLS2*, pENTR207-*SlSERK3A* and pENTR207-*SlSERK3B* were recombined into pEarleyGate103 generating C-terminal GFP-His-tag fusion constructs behind the 35S promoter. pENTR207-*SlSERK3A* and pENTR207-*SlSERK3B* were also recombined into pGWB14 generating C-terminal HA-tag fusion constructs behind the 35S promoter. All resulting constructs were sequence verified and transformed into *A. tumefaciens* strain GV3101.

The cytoplasmic domains (CD) of *Sl*SERK3A and *Sl*SERK3B were amplified from tomato cDNA using gene-specific primers (Table S2). Single point mutation variants of *Sl*SERK3A CD (D418N) and *Sl*SERK3B CD (D420N) were generated by PCR-based site-directed mutagenesis [65]. The amplified products were cloned into the pGEX4T-1 vector (Pharmacia) using *EcoR*I and *Not*I (NEB) to generate N-terminal GST fusion constructs. The resulting constructs were sequence verified.

### Recombinant protein purification and *in vitro* phosphorylation assays

Recombinant fusion proteins were produced in *Escherichia coli* strain BL21 (DE3). Bacteria were induced with 0.5 mM isopropyl-b-D-1-thiogalactopyranoside (IPTG) at 30°C for 4 h and extracted with lysis buffer containing 1× PBS, 1 M DTT, 0.1M ATP and 1 tablet protease inhibitor cocktail (Roche) for 10 ml buffer. The soluble fraction was used to enrich for the fusion proteins. GST-tagged fusion proteins were enriched using glutathione-agarose beads (BD Biosciences) according to the manufactures protocol. The eluted fusion proteins were adjusted to the same concentration in 1× PBS and 10% glycerol and incubated in the kinase buffer immediately. The *in vitro* phosphorylation of each kinase (1 µg) with [γ^−32^P] ATP was assayed as described earlier [34].

### RNA extraction and quantitative RT-PCR

RNA from leaves was extracted using TRIzol (Invitrogen) and treated with DNase I (New England Biolabs), while RNA from roots was extracted using hot phenol [59]. Five µg RNA was reverse-transcribed using SuperScript III reverse transcriptase (Invitrogen) and oligo-dT primer. For quantitative PCR, transcripts were amplified from 1 µl of a 5× diluted cDNA in a 15 µl reaction using gene-specific primers (Table S1) and iQ SYBR Green Supermix (Bio-Rad). The PCR amplification consisted of 3 min at 94°C, 40 cycles of 30 sec at 94°C, 30 sec at 58°C and 1 min at 72°C, 15 min at 72°C, followed by the generation of a dissociation curve. The generated threshold cycle (CT) was used to calculate transcript abundance relative to tomato *Ubi* gene as described previously [60]. DNase-treated RNA was used as template for control.

### Bacterial virulence assay

To prepare bacterial inoculum, a lawn of *Pseudomonas syringae* pv. *tomato (Pst)* DC3000 or *Pst* DC3000 *hrcC* was grown overnight at 30°C on King's medium B plates with appropriate antibiotics. Cells were collected from plates with 10 mM MgCl_2_ and adjusted to the desired colony-forming units (CFU)/ml. Five-week-old tomato VIGS plants were vacuum infiltrated with bacterial suspension (*Pst* DC3000 10^4^ CFU/ml and *Pst* DC3000 *hrcC* 5×10^4^ CFU/ml). To assess bacterial titer, five 1 cm^2^ leaf discs were harvested and ground in 1 ml 10 mM MgCl_2_, diluted and plated [61].

### Nematode virulence assay

*Meloidogyne incognita* was maintained on tomato cv. UC82B. Nematode eggs were extracted from infected roots in 0.5% NaOCl and eggs were hatched as described in Martinez de Illarduya et al. (2001) [62]. Three weeks after agroinfiltration, tomato roots were infected with freshly hatched 1000 infective-stage juveniles and maintained at 24°C. Six weeks later, roots were washed from soil particles, weighed and stained in 0.001% erioglaucine (Sigma). Individual roots were chopped into small pieces, mixed and egg masses were counted in two 10 g subsamples and the average calculated.

### Oxidative burst assay

For tomato, one leaf sample (two 2 mm^2^ per sample) from four 5-week-old plants was dissected with a sharp blade. For Arabidopsis, one leaf disc (4 mm diameter) from eight 4-week-old plants was sampled. Samples were floated overnight in sterile water and water was replaced with a solution of 1.7 µg/ml luminol (Sigma) and 10 µg/ml horseradish peroxidase (Sigma) containing 1 µM flg22. Luminescence was captured using a multiplate reader (BMG LUMIstar Galaxy Luminometer or BertholdTech TriStar).

### BL assays

For Arabidopsis root inhibition assays, sterilized seeds were vernalized at 4°C then sown on 1/2 MS medium supplemented with 1 nM epibrassinolide (BL) (Sigma) and 0.8% agar. Plates were incubated at 22°C, 16 h light and 8 h dark, 100 µEinstein/m^2^/s, for 9 days. Root length was measure for 50 seedlings per genotype and plotted as inhibition percentage compared with untreated roots [46].

For *SlCPD* expression analysis, tomato leaflets were syringe infiltrated with 10 µM BL 12 h before use.

### Transient expression in *N. benthamiana* for microscopy and immunoprecipitation

*Agrobacterium tumefaciens* containing constructs pEARLEYGATE103-*SlFLS2*, pEARLEYGATE103-*SlSERK3A*, pEARLEYGATE103-*SlSERK3B*, pCAMBIA-*AtBAK1*-mCherry, pGWB14-*SlSERK3A* and pGWB14-*SlSERK3B* were grown overnight in LB medium supplemented with appropriate antibiotics. Cultures were resuspended in 10 mM MgCl_2_, 10 mM MES, and 150 µM acetosyringone to a final OD_600_ = 0.2 to 0.5. After 3 h induction, cultures were infiltrated into 3-week-old *N. benthamiana* leaves using a needleless syringe.

### Microscopy

For localization, 35S-*SlSERK3A*-GFP or 35S-*SlSERK3B*-GFP (pEarleyGate103) and 35S-*AtBAK1*-mCherry (pCambia) proteins were transiently co-expressed in *N. benthamiana* leaves by agroinfiltration. Fluorescence was monitored 48 h later using a Leica SP2 Confocal microscope, with laser set at 488- and 563-nm to excite the GFP and mCherry, respectively, and images were collected through band emission filters at 500–530 and 600–630 nm, respectively.

For callose visualization, leaf discs were cleared using hot 95% ethanol, stained with 150 mM K2P04 (pH 9.5), 0.01% aniline blue for 2 h, and examined for UV fluorescence using Olympus BX51 microscope.

For H_2_0_2_ accumulation, leaf discs were vacuum infiltrating with 3,3′-diamaminobenzidine (DAB) as previously described [63]. Tissues were cleared with ethanol and examined under a bright-field microscope.

### Co-immunoprecipitation and immunoblot analysis

Leaf samples were processed as described earlier [28]. Samples were centrifuged at 13000 g for 20 min at 4°C, adjusted to 2 mg/ml total protein concentration, and pretreated with Protein *A*-agarose (Roach) for 3 to 4 h. Immunoprecipitations were performed on 1.5 ml total protein by adding anti-HA (Santa Cruz; 1∶100) or anti-GFP (Roach; 1∶100) overnight at 4°C. After incubation with 20 µl protein *A*-agarose at 4°C for 3 to 4 h, beads were washed 4 times with Tris-buffered saline (TBS) containing 0.5% (v/v) ND-40, immunoprecipitates were analyzed by immunobloting.

Samples were electrophoresed on 8% SDS-acrylamide gels, transferred onto nitrocellulose membranes (BIO-RAD), blocked, incubated overnight with primary antibody [anti-GFP (Roach) 1∶5000; anti-HA-HRP (Santa Cruz) 1∶2000], and washed in TBST (TBS with 0.1% (w/v) Tween-20). For anti-GFP, blots were incubated with a secondary antibody anti-mouse-HRP [(Santa Cruz) 1∶5000]. Signals were visualized using chemiluminescent substrate (Thermo Scientific) before exposure to X-ray film.

### Arabidopsis transgenic plants

*Agrobacterium tumefaciens* GV3101 containing pBAK1-*SlSERK3A* or pBAK1-*SlSERK3B* in pEARLEYGATE303 were transformed into the *bak1-4* mutant by the floral-dip method [64].

## Supporting Information

Figure S1 **The** ***SlSERK3*** **s have conserved** ***SERK*** **gene structure.** *SlSERK3A* and *SlSERK3B* gene structures with introns and exons shown as lines and boxes, respectively. Areas of the proteins coded by each exon are indicated beneath the boxes. SP, signal peptide; LRR, leucine-rich repeat; LRRNT, LRR N-terminal domain; SPP, proline-rich region; TM, transmembrane; & kinase subdomains (I–XI).
(PPTX)

Figure S2 **The characteristic domains of SERK proteins are conserved in** ***Sl*** **SERK3s.** The deduced amino acid sequence of tomato *Sl*SERK3s protein was aligned with the five Arabidopsis and two *Nicotiana benthamiana* SERK members. Conserved and most conserved amino acids residues are highlighted in black and grey, respectively. The protein domains are indicated below the sequences. Roman numerals indicate the position of the protein kinase catalytic subdomains. LRR, Leucine-rich repeat; LRRNT, LRR N-terminal domain. Double line in red indicate LRR C-terminal (LRRCT) domain. Single underline in black indicates the catalytic loop. Red star indicates the mutation to generate kinase dead mutants (D to N).
(PPTX)

Figure S3 **Gene fragments used in VIGS.** (**A**) Position of TRV-*Sl*FLS2 VIGS fragment used for silencing relative to the full-length open reading frame (ORF). (**B**), Upper panel, position of TRV-*Sl*SERK3A *VIGS fragment* used for silencing relative to the ORF. Lower panel, line-up of the TRV-*Sl*SERK3A fragment with the corresponding region in *SlSERK3B*. (**C**) Upper panel, position of TRV-*SlSERK3B VIGS fragment* used for silencing relative to the ORF. Lower panel, line-up of the TRV-*Sl*SERK3B fragment with the corresponding region in *SlSERK3A*. (**D**) Upper panel, position of TRV-*Sl*SERK3AB VIGS fragment (originating from *SlSERK3B*) used for co-silencing *SlSERK3A* and *SlSERK3B* relative to their ORF. Lower panel, line-up of the TRV-*Sl*SERK3AB fragment with the corresponding regions in *SlSERK3A* and *SlSERK3B*.
(PPTX)

Figure S4 **Silencing individually** ***SlSERK3A*** **or** ***SlSERK3B*** **does not result in cell death.** (**A**) Transcript levels of VIGS-silenced genes were evaluated using qRT-PCR. Additional samples (to those presented in Figure 3A) of tomato cv. Moneymaker plants (used in the bacterial screens) treated with TRV empty vector (TRV), TRV-*Sl*SERK3A, TRV-*Sl*SERK3B, and TRV-*Sl*SERK3AB were evaluated. Expression was normalized against *UBI3*. Values are average ± SE of three technical replicates. *P<0.05 significant difference from TRV (two-sample *t*-test). (**B**) Tomato cv. Moneymaker leaflets from plants silenced with the indicated TRV constructs. Photos were taken 3 weeks after TRV treatment. (**C**) Aniline blue-stained tomato leaf discs. No callose deposits were detected in leaflets silenced for either *SlSERK3A* or *SlSERK3B*. Leaves treated with 1 mM flg22 for 24 h were used as control.
(PPTX)

Figure S5 **Co-silencing** ***SlSERK3A*** **and** ***SlSERK3B*** **result in cell death and reduced BR sensitivity.** (**A**) DAB-stained tomato leaf discs. Leaflets of tomato cv. Moneymaker plants co-silenced for *SlSERK3A* and *SlSERK3B* showing cell death and TRV empty vector (TRV) control were evaluated for H_2_O_2_ accumulation. (**B**) Leaflets of tomato cv. Moneymaker plants silenced for *SlSERK3A*, *SlSERK3B* or co-silenced and TRV control were evaluated for BR-sensitivity. Leaflets were infiltrated with 10 µM BL 12 h before use. Transcript levels of VIGS-silenced genes and *SlCPD* were evaluated using qRT-PCR normalized against *UBI3*. Values represent the average and ± SE of three biological replicates. *P<0.05 and **P<0.01 indicate significant difference from the respective – BL control (two-sample *t*-test). This experiment was repeated twice with similar results.
(PPTX)

Figure S6 ***SlFLS2*** **transcript levels in TRV-** ***SlFLS2*** **treated plants.** Transcript levels were evaluated in leaflets of tomato cv. Moneymaker silenced for *SlFLS2* and TRV empty vector (TRV) control using qRT-PCR. Expression was normalized against *UBI3*. Four independent samples were analyzed per construct. Values are average ± SE of three technical replicates. *P<0.05 significant difference from TRV (two-sample *t*-test).
(PPTX)

Figure S7 ***SlSERK3A*** **and** ***SlSERK3B*** **transcript levels in silenced roots and root weight.** (**A**) Transcript levels of VIGS-silenced genes were evaluated using qRT-PCR. Tomato cv. Moneymaker plants, treated with TRV empty vector (TRV), TRV-*Sl*SERK3A, or TRV-*Sl*SERK3B, were evaluated. Expression was normalized against *UBI3*. A subsample from six different roots was analyzed per construct. This experiment was performed twice and data from both experiments are presented. Values are average ± SE of three technical replicates. *P<0.05 significant difference from TRV (two-sample t-test). (**B**) Root weight of RKN infected plants. Values are average (±) SE (n = 9) from a single experiment. No significance difference (ANOVA Tukey's HSD test) was observed in root weight.
(PPTX)

Figure S8 ***SlSERK3A*** **and** ***SlSERK3B*** **complemented the Arabidopsis** ***bak1-4*** **mutant BR-induced root length inhibition.** Transgenic *bak1-4* plants expressing pBAK1-*SlSERK3A (bak1-4 SlSERK3A)* or pBAK1-*SlSERK3B (bak1-4 SlSERK3B)* and *bak1-4* mutant plants were evaluated for root growth. Nine-day-old Arabidopsis seedlings root grown on medium with (right panel) or without (left panel) 1 nM BL.
(PPTX)

Table S1 **List of primers used in qPCR.**
(DOC)

Table S2 **List of primers used in cloning.**
(DOC)
