# Supplementary material for: The Tomato Leucine-Rich Repeat Receptor-Like Kinases SlSERK3A and SlSERK3B Have Overlapping Functions in Bacterial and Nematode Innate Immunity
Source: PLoS One. 2014 Mar 27;9(3):e93302. doi: 10.1371/journal.pone.0093302 (PMC3968124; doi:10.1371/journal.pone.0093302)
Supplement: Figure S1 — The SlSERK3 s have conserved SERK gene structure. SlSERK3A and SlSERK3B gene structures with introns and exons shown as lines and boxes, respectively. Areas of the proteins coded by each exon are indicated beneath the boxes. SP, signal peptide; LRR, leucine-rich repeat; LRRNT, LRR N-terminal domain; SPP, proline-rich region; TM, transmembrane; & kinase subdomains (I–XI). (PPTX) [file pone.0093302.s001.pptx]

## Slide 1
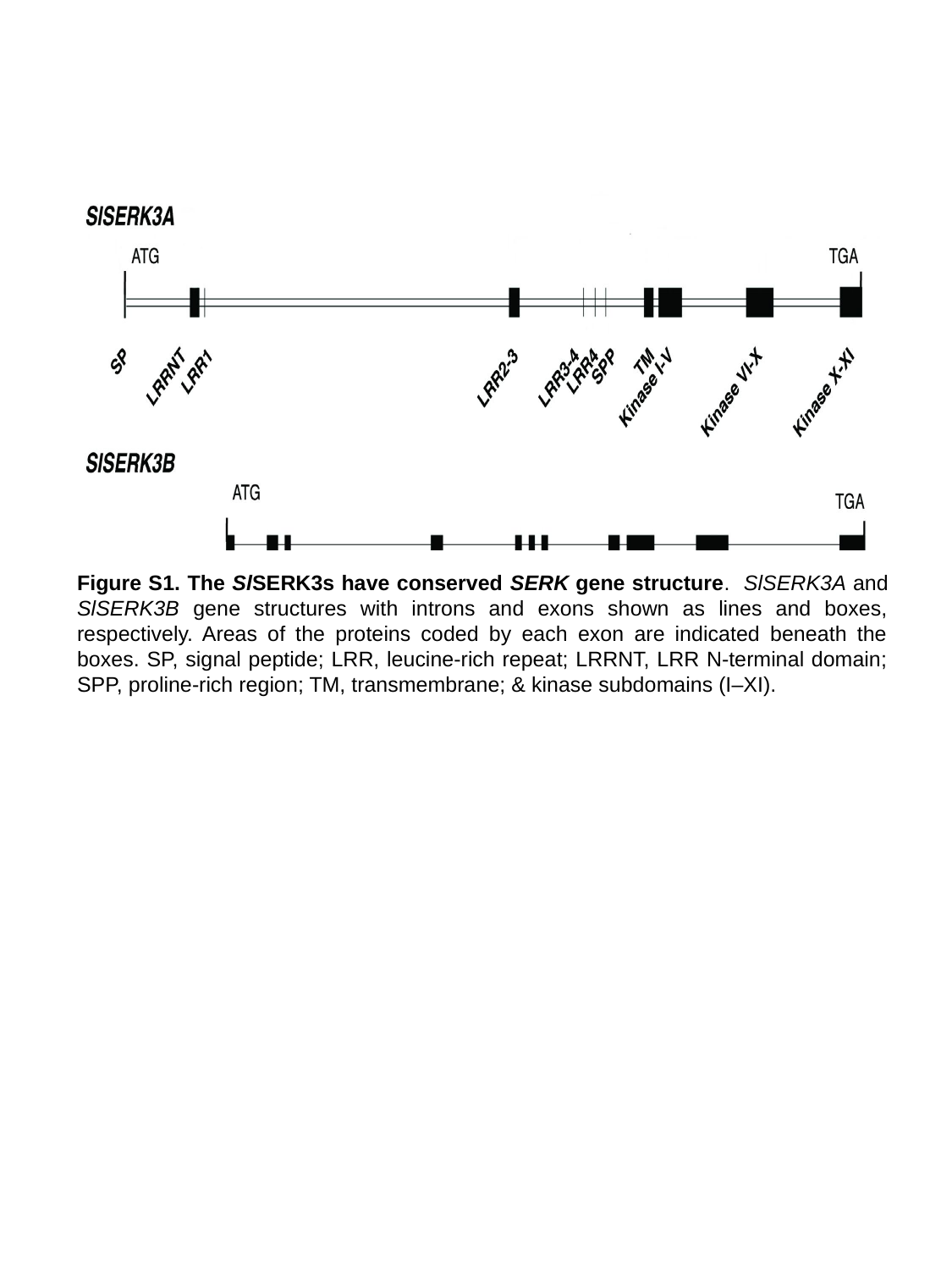

Figure S1. The SlSERK3s have conserved SERK gene structure. SlSERK3A and SlSERK3B gene structures with introns and exons shown as lines and boxes, respectively. Areas of the proteins coded by each exon are indicated beneath the boxes. SP, signal peptide; LRR, leucine-rich repeat; LRRNT, LRR N-terminal domain; SPP, proline-rich region; TM, transmembrane; & kinase subdomains (I–XI).
