# Supplementary material for: The Tomato Leucine-Rich Repeat Receptor-Like Kinases SlSERK3A and SlSERK3B Have Overlapping Functions in Bacterial and Nematode Innate Immunity
Source: PLoS One. 2014 Mar 27;9(3):e93302. doi: 10.1371/journal.pone.0093302 (PMC3968124; doi:10.1371/journal.pone.0093302)
Supplement: Figure S2 — The characteristic domains of SERK proteins are conserved in Sl SERK3s. The deduced amino acid sequence of tomato SlSERK3s protein was aligned with the five Arabidopsis and two Nicotiana benthamiana SERK members. Conserved and most conserved amino acids residues are highlighted in black and grey, respectively. The protein domains are indicated below the sequences. Roman numerals indicate the position of the protein kinase catalytic subdomains. LRR, Leucine-rich repeat; LRRNT, LRR N-terminal domain. Double line in red indicate LRR C-terminal (LRRCT) domain. Single underline in black indicates the catalytic loop. Red star indicates the mutation to generate kinase dead mutants (D to N). (PPTX) [file pone.0093302.s002.pptx]

## Slide 1
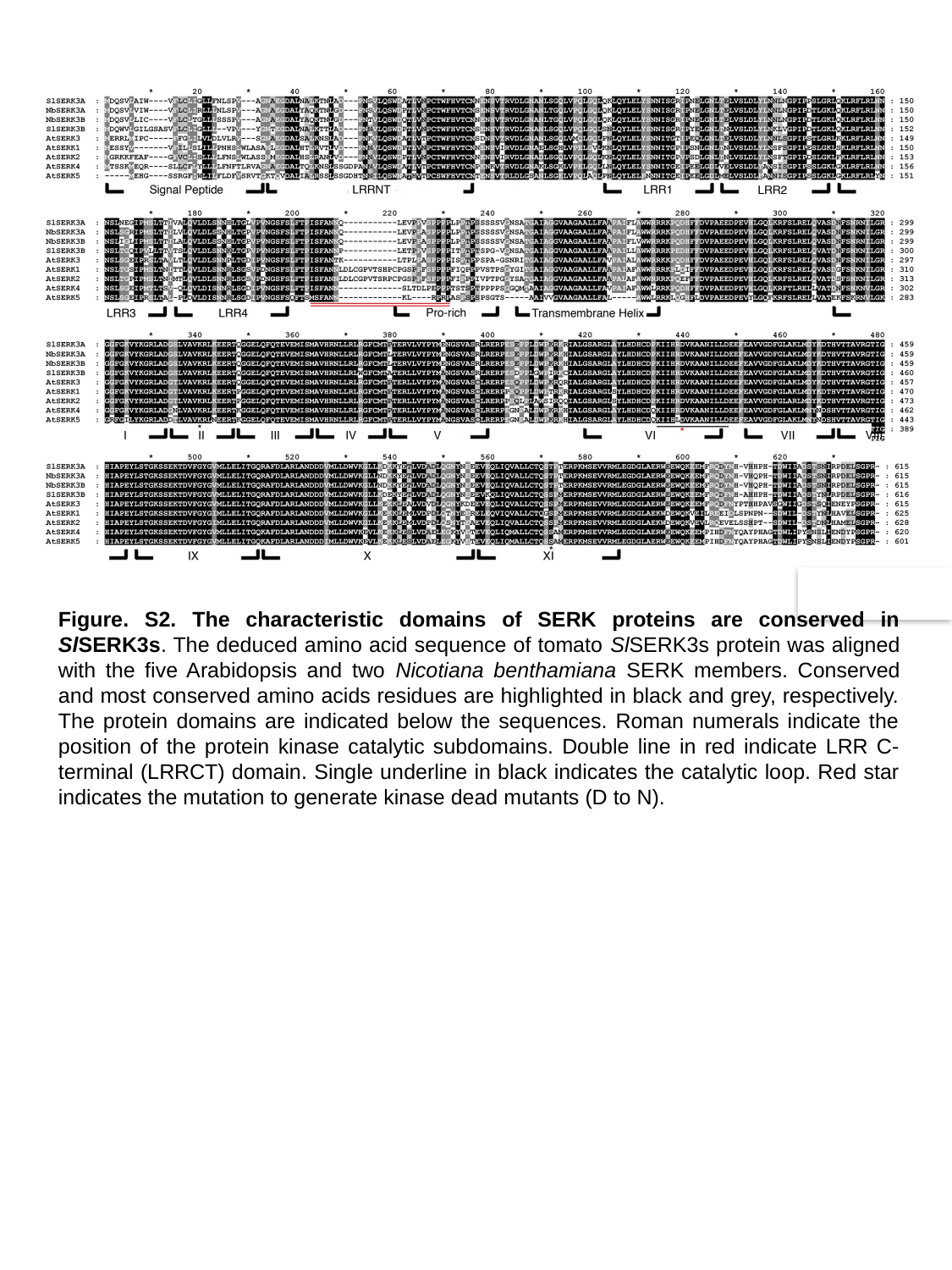

Figure. S2. The characteristic domains of SERK proteins are conserved in SlSERK3s. The deduced amino acid sequence of tomato SlSERK3s protein was aligned with the five Arabidopsis and two Nicotiana benthamiana SERK members. Conserved and most conserved amino acids residues are highlighted in black and grey, respectively. The protein domains are indicated below the sequences. Roman numerals indicate the position of the protein kinase catalytic subdomains. Double line in red indicate LRR C-terminal (LRRCT) domain. Single underline in black indicates the catalytic loop. Red star indicates the mutation to generate kinase dead mutants (D to N).
