# Supplementary material for: The Tomato Leucine-Rich Repeat Receptor-Like Kinases SlSERK3A and SlSERK3B Have Overlapping Functions in Bacterial and Nematode Innate Immunity
Source: PLoS One. 2014 Mar 27;9(3):e93302. doi: 10.1371/journal.pone.0093302 (PMC3968124; doi:10.1371/journal.pone.0093302)
Supplement: Figure S3 — Gene fragments used in VIGS. (A) Position of TRV-SlFLS2 VIGS fragment used for silencing relative to the full-length open reading frame (ORF). (B), Upper panel, position of TRV-SlSERK3A VIGS fragment used for silencing relative to the ORF. Lower panel, line-up of the TRV-SlSERK3A fragment with the corresponding region in SlSERK3B. (C) Upper panel, position of TRV-SlSERK3B VIGS fragment used for silencing relative to the ORF. Lower panel, line-up of the TRV-SlSERK3B fragment with the corresponding region in SlSERK3A. (D) Upper panel, position of TRV-SlSERK3AB VIGS fragment (originating from SlSERK3B) used for co-silencing SlSERK3A and SlSERK3B relative to their ORF. Lower panel, line-up of the TRV-SlSERK3AB fragment with the corresponding regions in SlSERK3A and SlSERK3B. (PPTX) [file pone.0093302.s003.pptx]

## Slide 1
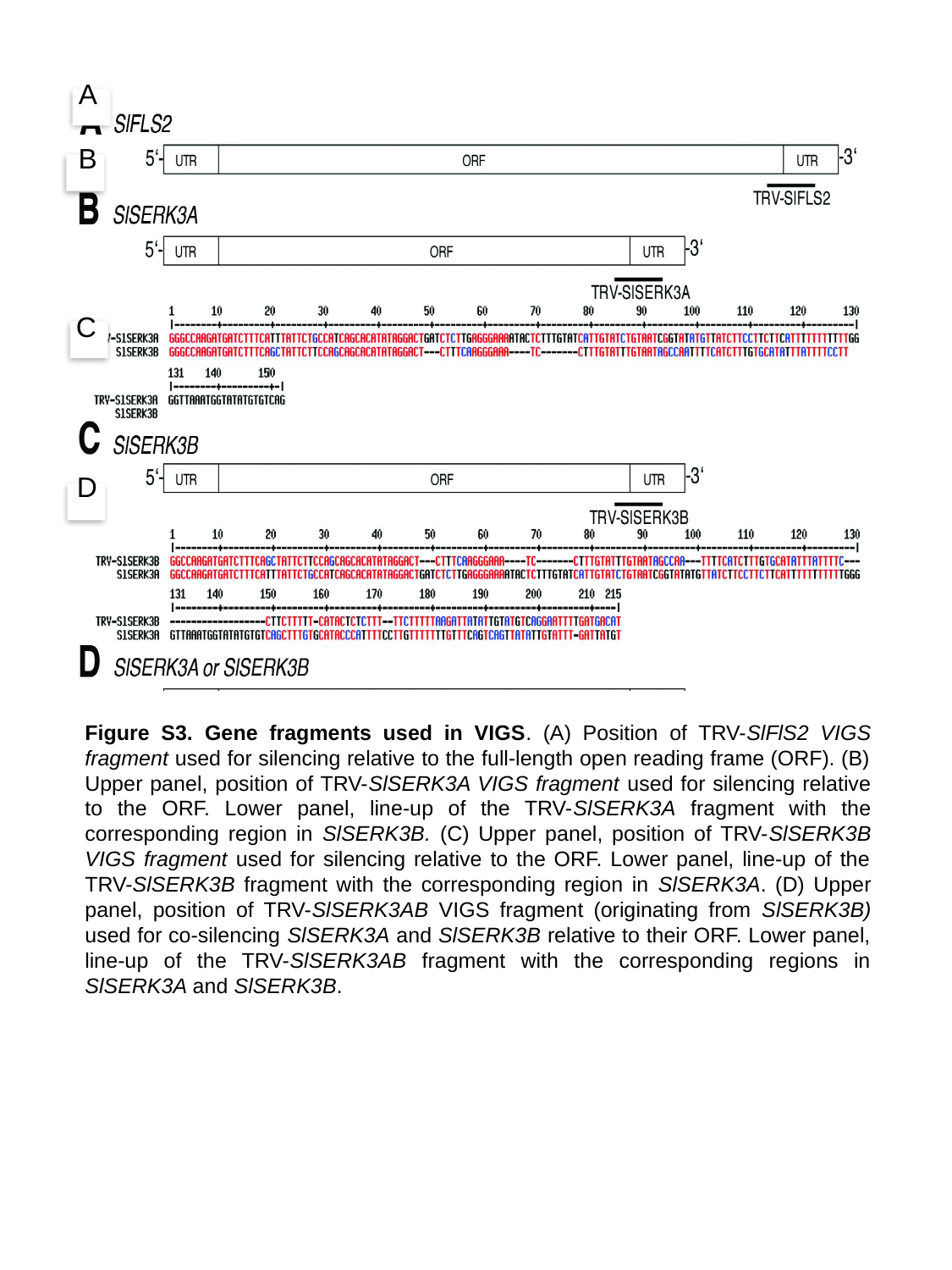

A
B
C
D
Figure S3. Gene fragments used in VIGS. (A) Position of TRV-SlFlS2 VIGS fragment used for silencing relative to the full-length open reading frame (ORF). (B) Upper panel, position of TRV-SlSERK3A VIGS fragment used for silencing relative to the ORF. Lower panel, line-up of the TRV-SlSERK3A fragment with the corresponding region in SlSERK3B. (C) Upper panel, position of TRV-SlSERK3B VIGS fragment used for silencing relative to the ORF. Lower panel, line-up of the TRV-SlSERK3B fragment with the corresponding region in SlSERK3A. (D) Upper panel, position of TRV-SlSERK3AB VIGS fragment (originating from SlSERK3B) used for co-silencing SlSERK3A and SlSERK3B relative to their ORF. Lower panel, line-up of the TRV-SlSERK3AB fragment with the corresponding regions in SlSERK3A and SlSERK3B.
