# Supplementary material for: The Tomato Leucine-Rich Repeat Receptor-Like Kinases SlSERK3A and SlSERK3B Have Overlapping Functions in Bacterial and Nematode Innate Immunity
Source: PLoS One. 2014 Mar 27;9(3):e93302. doi: 10.1371/journal.pone.0093302 (PMC3968124; doi:10.1371/journal.pone.0093302)
Supplement: Figure S4 — Silencing individually SlSERK3A or SlSERK3B does not result in cell death. (A) Transcript levels of VIGS-silenced genes were evaluated using qRT-PCR. Additional samples (to those presented in Figure 3A) of tomato cv. Moneymaker plants (used in the bacterial screens) treated with TRV empty vector (TRV), TRV-SlSERK3A, TRV-SlSERK3B, and TRV-SlSERK3AB were evaluated. Expression was normalized against UBI3. Values are average ± SE of three technical replicates. *P<0.05 significant difference from TRV (two-sample t-test). (B) Tomato cv. Moneymaker leaflets from plants silenced with the indicated TRV constructs. Photos were taken 3 weeks after TRV treatment. (C) Aniline blue-stained tomato leaf discs. No callose deposits were detected in leaflets silenced for either SlSERK3A or SlSERK3B. Leaves treated with 1 mM flg22 for 24 h were used as control. (PPTX) [file pone.0093302.s004.pptx]

## Slide 1
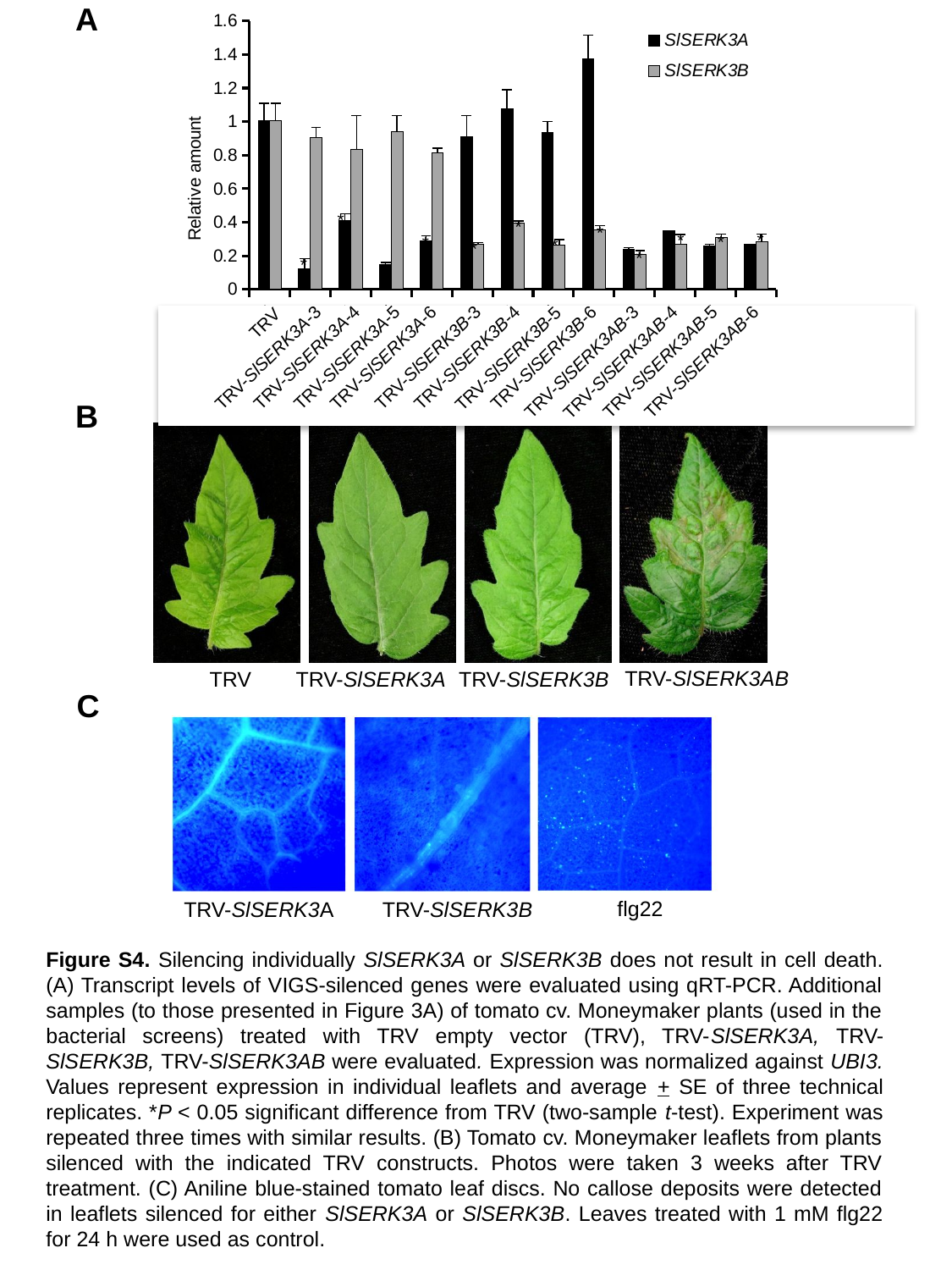

A
### Chart
| Category | SlSERK3A | SlSERK3B |
|---|---|---|
| TRV | 1.005409967339339 | 1.005409967339339 |
| S3A-3 | 0.11966943295319 | 0.90341637208928 |
| S3A-4 | 0.408323327645911 | 0.836272618750505 |
| S3A-5 | 0.149008131211694 | 0.938080669547548 |
| S3A-6 | 0.28872819389508 | 0.812740256575233 |
| S3B-3 | 0.909924510869065 | 0.268104299488653 |
| S3B-4 | 1.077571721963784 | 0.392527669902858 |
| S3B-5 | 0.93527528164806 | 0.261307197201694 |
| S3B-6 | 1.373430489927658 | 0.354403059660412 |
| S3AB-3 | 0.238853776742355 | 0.208933716503036 |
| S3AB-4 | 0.349492483544755 | 0.270612938376237 |
| S3AB-5 | 0.258971682817036 | 0.30852578322124 |
| S3AB-6 | 0.267943365634074 | 0.281567612788713 |*
*
*
*
*
*
*
*
*
*
*
*
*
*
 *
 *
TRV
TRV-SlSERK3A-4
TRV-SlSERK3B-3
TRV-SlSERK3A-6
TRV-SlSERK3A-3
TRV-SlSERK3B-4
TRV-SlSERK3B-6
TRV-SlSERK3A-5
TRV-SlSERK3B-5
TRV-SlSERK3AB-5
TRV-SlSERK3AB-6
TRV-SlSERK3AB-3
TRV-SlSERK3AB-4
B
TRV-SlSERK3AB
TRV-SlSERK3A
TRV
TRV-SlSERK3B
C
flg22
TRV-SlSERK3A
TRV-SlSERK3B
Figure S4. Silencing individually SlSERK3A or SlSERK3B does not result in cell death. (A) Transcript levels of VIGS-silenced genes were evaluated using qRT-PCR. Additional samples (to those presented in Figure 3A) of tomato cv. Moneymaker plants (used in the bacterial screens) treated with TRV empty vector (TRV), TRV-SlSERK3A, TRV-SlSERK3B, TRV-SlSERK3AB were evaluated. Expression was normalized against UBI3. Values represent expression in individual leaflets and average + SE of three technical replicates. *P < 0.05 significant difference from TRV (two-sample t-test). Experiment was repeated three times with similar results. (B) Tomato cv. Moneymaker leaflets from plants silenced with the indicated TRV constructs. Photos were taken 3 weeks after TRV treatment. (C) Aniline blue-stained tomato leaf discs. No callose deposits were detected in leaflets silenced for either SlSERK3A or SlSERK3B. Leaves treated with 1 mM flg22 for 24 h were used as control.
