# Supplementary material for: The Tomato Leucine-Rich Repeat Receptor-Like Kinases SlSERK3A and SlSERK3B Have Overlapping Functions in Bacterial and Nematode Innate Immunity
Source: PLoS One. 2014 Mar 27;9(3):e93302. doi: 10.1371/journal.pone.0093302 (PMC3968124; doi:10.1371/journal.pone.0093302)
Supplement: Figure S5 — Co-silencing SlSERK3A and SlSERK3B result in cell death and reduced BR sensitivity. (A) DAB-stained tomato leaf discs. Leaflets of tomato cv. Moneymaker plants co-silenced for SlSERK3A and SlSERK3B showing cell death and TRV empty vector (TRV) control were evaluated for H2O2 accumulation. (B) Leaflets of tomato cv. Moneymaker plants silenced for SlSERK3A, SlSERK3B or co-silenced and TRV control were evaluated for BR-sensitivity. Leaflets were infiltrated with 10 µM BL 12 h before use. Transcript levels of VIGS-silenced genes and SlCPD were evaluated using qRT-PCR normalized against UBI3. Values represent the average and ± SE of three biological replicates. *P<0.05 and **P<0.01 indicate significant difference from the respective – BL control (two-sample t-test). This experiment was repeated twice with similar results. (PPTX) [file pone.0093302.s005.pptx]

## Slide 1
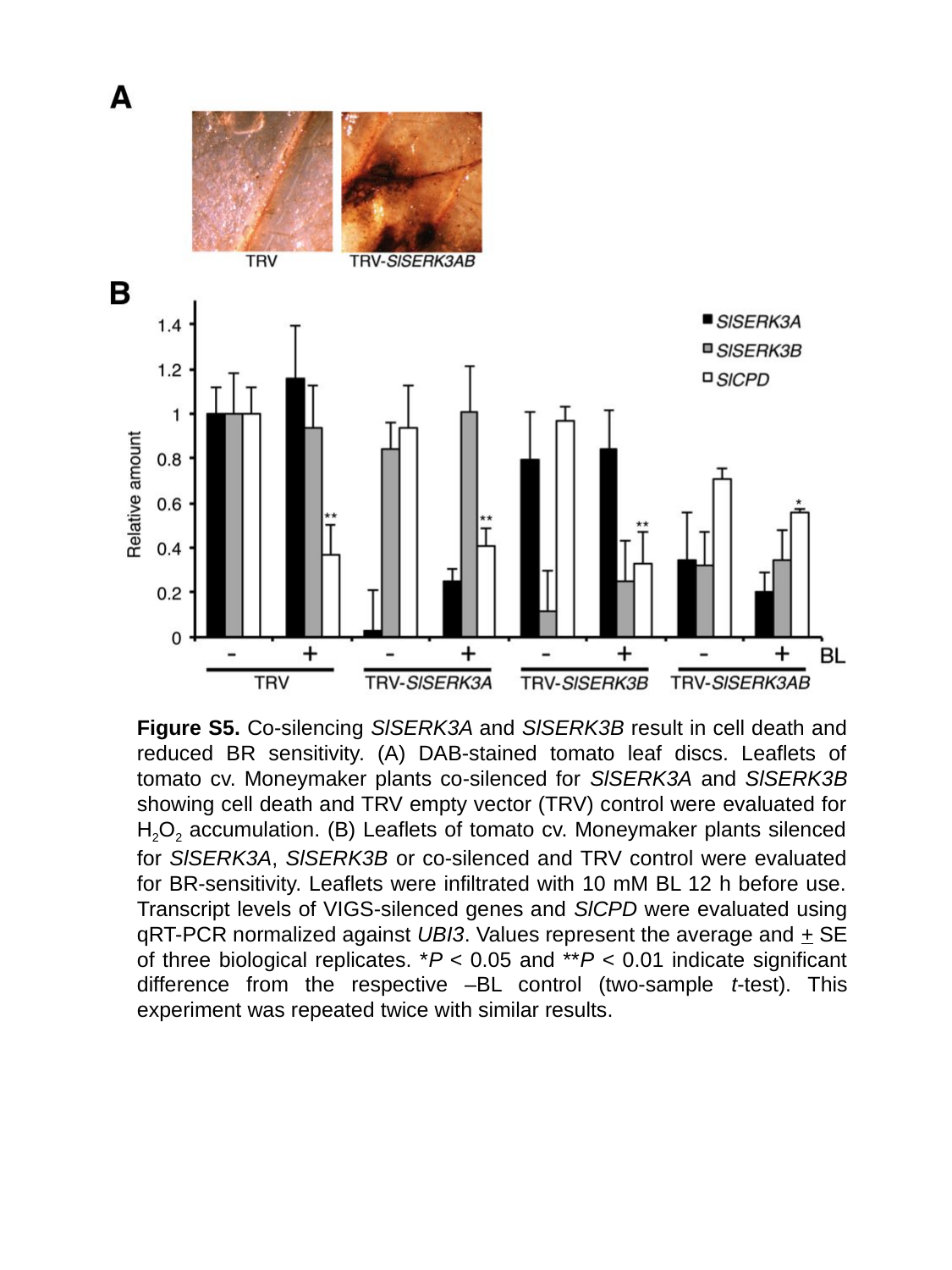

Figure S5. Co-silencing SlSERK3A and SlSERK3B result in cell death and reduced BR sensitivity. (A) DAB-stained tomato leaf discs. Leaflets of tomato cv. Moneymaker plants co-silenced for SlSERK3A and SlSERK3B showing cell death and TRV empty vector (TRV) control were evaluated for H2O2 accumulation. (B) Leaflets of tomato cv. Moneymaker plants silenced for SlSERK3A, SlSERK3B or co-silenced and TRV control were evaluated for BR-sensitivity. Leaflets were infiltrated with 10 mM BL 12 h before use. Transcript levels of VIGS-silenced genes and SlCPD were evaluated using qRT-PCR normalized against UBI3. Values represent the average and + SE of three biological replicates. *P < 0.05 and **P < 0.01 indicate significant difference from the respective –BL control (two-sample t-test). This experiment was repeated twice with similar results.
