# Supplementary material for: The Tomato Leucine-Rich Repeat Receptor-Like Kinases SlSERK3A and SlSERK3B Have Overlapping Functions in Bacterial and Nematode Innate Immunity
Source: PLoS One. 2014 Mar 27;9(3):e93302. doi: 10.1371/journal.pone.0093302 (PMC3968124; doi:10.1371/journal.pone.0093302)
Supplement: Figure S6 — SlFLS2 transcript levels in TRV- SlFLS2 treated plants. Transcript levels were evaluated in leaflets of tomato cv. Moneymaker silenced for SlFLS2 and TRV empty vector (TRV) control using qRT-PCR. Expression was normalized against UBI3. Four independent samples were analyzed per construct. Values are average ± SE of three technical replicates. *P<0.05 significant difference from TRV (two-sample t-test). (PPTX) [file pone.0093302.s006.pptx]

## Slide 1
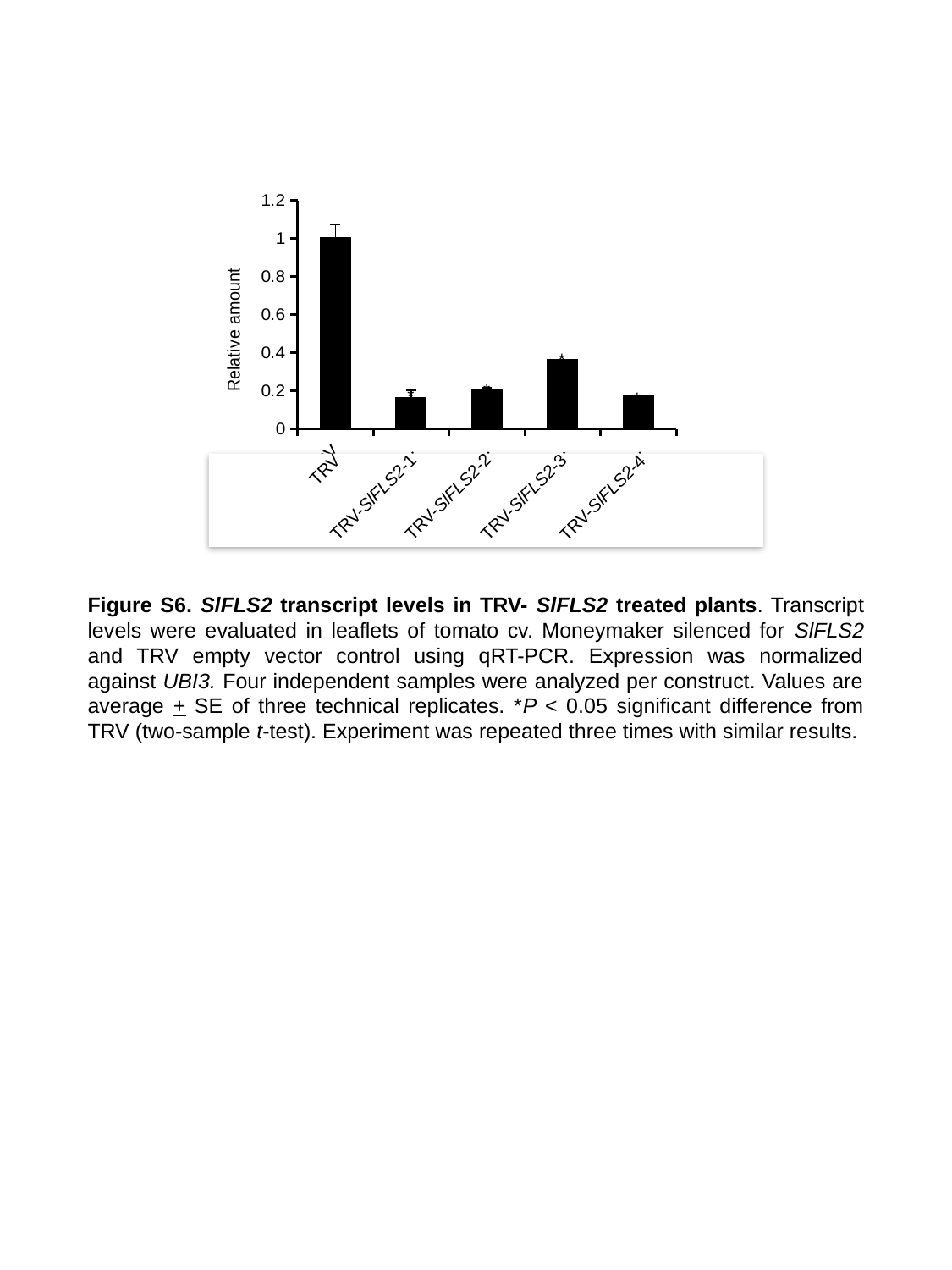

### Chart
| Category | |
|---|---|
| TRV | 1.002403227036552 |
| TRV-SlFLS2-1 | 0.164031549544529 |
| TRV-SlFLS2-2 | 0.210350369956545 |
| TRV-SlFLS2-3 | 0.366021423986406 |
| TRV-SlFLS2-4 | 0.176776695296637 |
TRV-SlFLS2-2
TRV-SlFLS2-3
TRV
TRV-SlFLS2-1
TRV-SlFLS2-4
*
*
*
*
Figure S6. SlFLS2 transcript levels in TRV- SlFLS2 treated plants. Transcript levels were evaluated in leaflets of tomato cv. Moneymaker silenced for SlFLS2 and TRV empty vector control using qRT-PCR. Expression was normalized against UBI3. Four independent samples were analyzed per construct. Values are average + SE of three technical replicates. *P < 0.05 significant difference from TRV (two-sample t-test). Experiment was repeated three times with similar results.
