# Supplementary material for: The Tomato Leucine-Rich Repeat Receptor-Like Kinases SlSERK3A and SlSERK3B Have Overlapping Functions in Bacterial and Nematode Innate Immunity
Source: PLoS One. 2014 Mar 27;9(3):e93302. doi: 10.1371/journal.pone.0093302 (PMC3968124; doi:10.1371/journal.pone.0093302)
Supplement: Figure S7 — SlSERK3A and SlSERK3B transcript levels in silenced roots and root weight. (A) Transcript levels of VIGS-silenced genes were evaluated using qRT-PCR. Tomato cv. Moneymaker plants, treated with TRV empty vector (TRV), TRV-SlSERK3A, or TRV-SlSERK3B, were evaluated. Expression was normalized against UBI3. A subsample from six different roots was analyzed per construct. This experiment was performed twice and data from both experiments are presented. Values are average ± SE of three technical replicates. *P<0.05 significant difference from TRV (two-sample t-test). (B) Root weight of RKN infected plants. Values are average (±) SE (n = 9) from a single experiment. No significance difference (ANOVA Tukey's HSD test) was observed in root weight. (PPTX) [file pone.0093302.s007.pptx]

## Slide 1
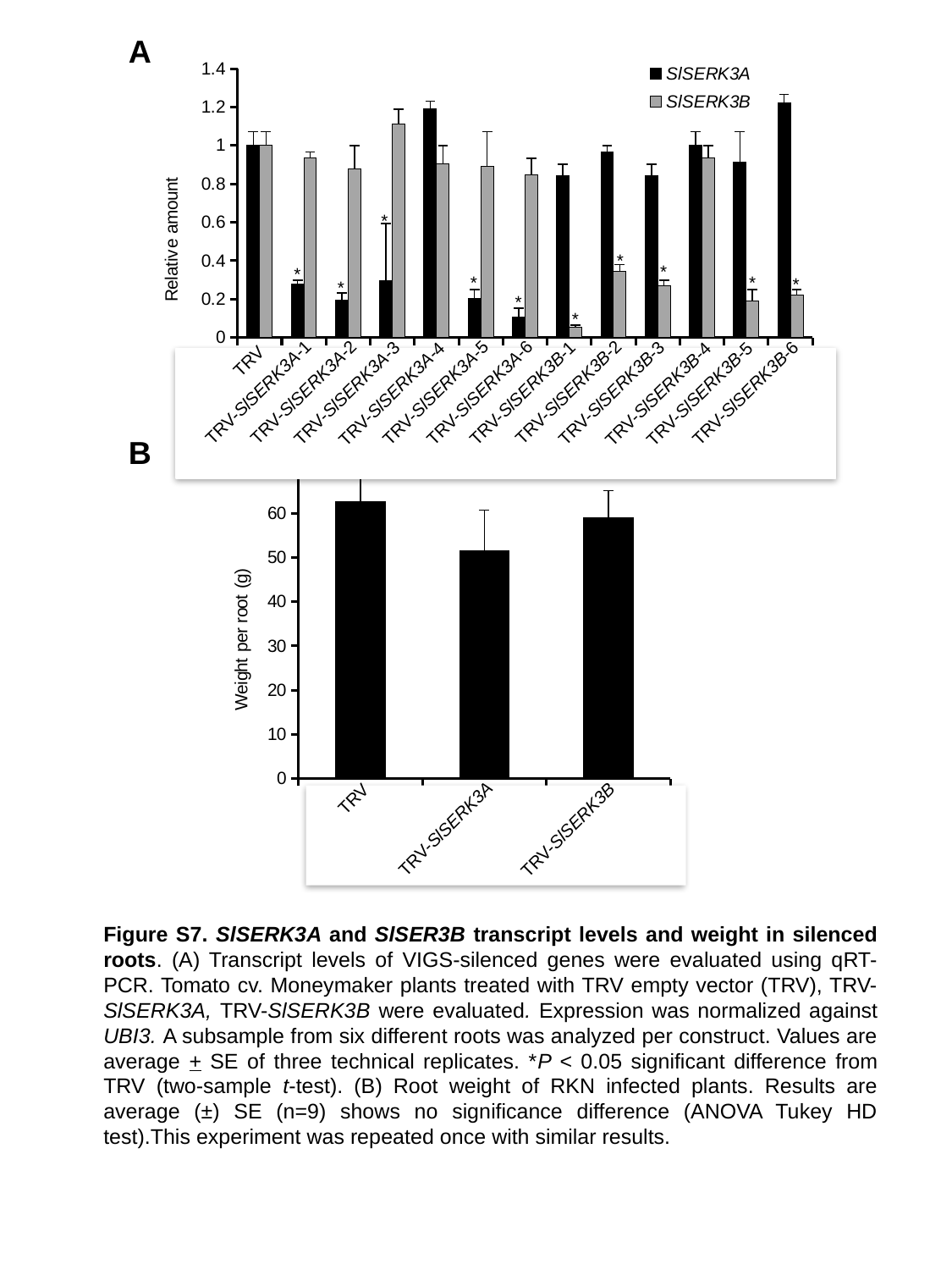

A
### Chart
| Category | SlSERK3A | SlSERK3B |
|---|---|---|
| TRV | 1.002403227036551 | 1.002403227036551 |
| SERK3A-1 | 0.278059004855512 | 0.933593395767836 |
| SERK3A-2 | 0.193575649776158 | 0.878929141627599 |
| SERK3A-3 | 0.297572669735072 | 1.11223601942205 |
| SERK3A-4 | 1.189921384170975 | 0.906126198178117 |
| SERK3A-5 | 0.201946525834057 | 0.889440121861422 |
| SERK3A-6 | 0.108196525834057 | 0.845445637396003 |
| SERK3B-1 | 0.842917280253789 | 0.0552283678691934 |
| SERK3B-2 | 0.966516495768404 | 0.343357622481915 |
| SERK3B-3 | 0.842917280253789 | 0.269392930490945 |
| SERK3B-4 | 1.002403227036548 | 0.935275281648062 |
| SERK3B-5 | 0.914815872895746 | 0.191985841408519 |
| SERK3B-6 | 1.219825556881316 | 0.2197322854069 |*
*
*
*
*
*
*
*
*
*
TRV
TRV-SlSERK3A-1
TRV-SlSERK3A-2
TRV-SlSERK3B-2
TRV-SlSERK3A-5
TRV-SlSERK3A-6
TRV-SlSERK3B-3
TRV-SlSERK3A-3
TRV-SlSERK3B-1
TRV-SlSERK3B-6
TRV-SlSERK3A-4
TRV-SlSERK3B-5
TRV-SlSERK3B-4
B
### Chart
| Category | |
|---|---|
| TRV | 62.60333333333334 |
| TRV-SERK3A | 51.6 |
| TRV-SERK3B | 58.94444444444426 |
TRV
TRV-SlSERK3A
TRV-SlSERK3B
Figure S7. SlSERK3A and SlSER3B transcript levels and weight in silenced roots. (A) Transcript levels of VIGS-silenced genes were evaluated using qRT-PCR. Tomato cv. Moneymaker plants treated with TRV empty vector (TRV), TRV-SlSERK3A, TRV-SlSERK3B were evaluated. Expression was normalized against UBI3. A subsample from six different roots was analyzed per construct. Values are average + SE of three technical replicates. *P < 0.05 significant difference from TRV (two-sample t-test). (B) Root weight of RKN infected plants. Results are average (±) SE (n=9) shows no significance difference (ANOVA Tukey HD test).This experiment was repeated once with similar results.
