# Supplementary material for: The Tomato Leucine-Rich Repeat Receptor-Like Kinases SlSERK3A and SlSERK3B Have Overlapping Functions in Bacterial and Nematode Innate Immunity
Source: PLoS One. 2014 Mar 27;9(3):e93302. doi: 10.1371/journal.pone.0093302 (PMC3968124; doi:10.1371/journal.pone.0093302)
Supplement: Figure S8 — SlSERK3A and SlSERK3B complemented the Arabidopsis bak1-4 mutant BR-induced root length inhibition. Transgenic bak1-4 plants expressing pBAK1-SlSERK3A (bak1-4 SlSERK3A) or pBAK1-SlSERK3B (bak1-4 SlSERK3B) and bak1-4 mutant plants were evaluated for root growth. Nine-day-old Arabidopsis seedlings root grown on medium with (right panel) or without (left panel) 1 nM BL. (PPTX) [file pone.0093302.s008.pptx]

## Slide 1
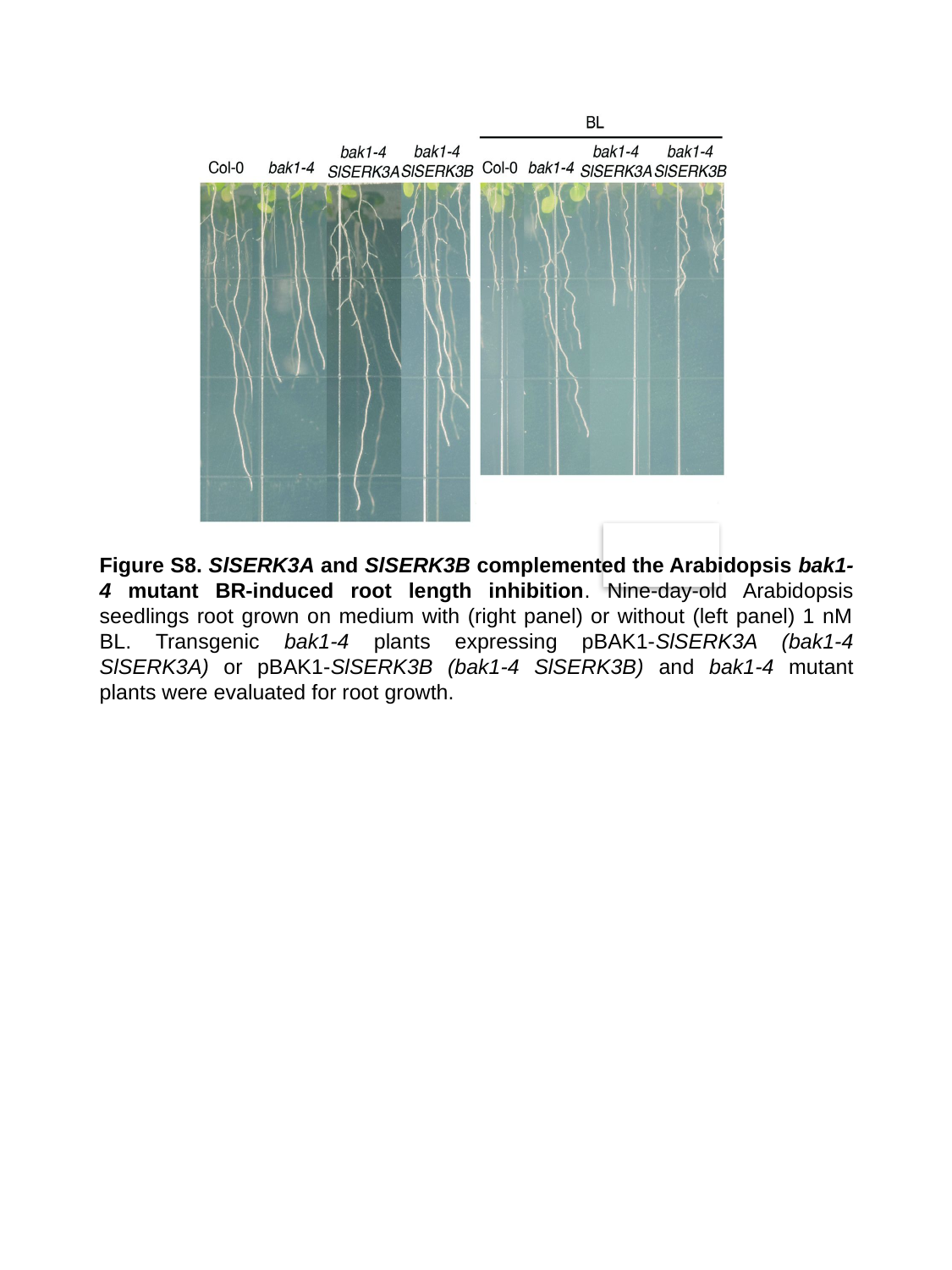

Figure S8. SlSERK3A and SlSERK3B complemented the Arabidopsis bak1-4 mutant BR-induced root length inhibition. Nine-day-old Arabidopsis seedlings root grown on medium with (right panel) or without (left panel) 1 nM BL. Transgenic bak1-4 plants expressing pBAK1-SlSERK3A (bak1-4 SlSERK3A) or pBAK1-SlSERK3B (bak1-4 SlSERK3B) and bak1-4 mutant plants were evaluated for root growth.
