# Supplementary material for: The Tomato Leucine-Rich Repeat Receptor-Like Kinases SlSERK3A and SlSERK3B Have Overlapping Functions in Bacterial and Nematode Innate Immunity
Source: PLoS One. 2014 Mar 27;9(3):e93302. doi: 10.1371/journal.pone.0093302 (PMC3968124; doi:10.1371/journal.pone.0093302)
Supplement: Table S1 — List of primers used in qPCR. (DOC) [file pone.0093302.s009.doc]

**Supplementary Table S1.** List of primers used in qPCR

| **Gene** | **Primer name** | **Sequence (5'-3')*** | **Reference** |
| --- | --- | --- | --- |
| *SlSERK3A* | LeSERK3qPCR-1029-F | TGTACACCGAAACCTACTTCGTTTA | Mantelin *et al.* 2011 |
|  | LeSERK3qPCR-1407-R | ACCAGTAGATAAATATTCAGGGGCA | Mantelin *et al.* 2011 |
| *SlSERK3B* | nSlSERKqPCR-For | CCATGCTCACCATCCTCAC | Mantelin *et al.* 2011 |
|  | nSlSERKqPCR-Rev | TGTGCTGCTGGAAGAATAGC | Mantelin *et al.* 2011 |
| *SlFLS2* | SlFLS2 qPCR-F | GGATCATACTTGCAGCACTTGG | This study |
|  | SlFLS2 qPCR-R | ATCTCTGGAGGCTCAGTGCTG | This study |
| *SlUbi3* | Ubi3-qRT-For | GTGTGGGCTCACCTACGTTT | Bhattarai *et al.* 2010 |
|  | Ubi3-qRT-Rev | CCGTTCATTCGACAAAAAGAA | Bhattarai *et al.* 2010 |
| *SlCPD* | SlCPD-qPCR-F | CTTCTCTCCGAGCTGTTCATCTAG | This Study |
|  | SlCPD-qPCR-R | GAAGGAAAACAGAGAGTTCCACTC | This Study |
| *SlWRKY28* | WRKY28-F | ACAGATGCAGCTACCTCATCCTCA | Kim et al. 2009 |
|  | WRKY28-R | GTGCTCAAAGCCTCATGGTTCTTG | Kim et al. 2009 |
| *SlPti5* | PTI5-F | ATTCGCGATTCGGCTAGACATGGT | Kim et al. 2009 |
|  | PTI5-R | AGTAGTGCCTTAGCACCTCGCATT | Kim et al. 2009 |
| *BAK1* | BAK1-F | AAAGACGAAGAAGTGGAGCAGC | This Study |
|  | BAK1-R | GCCACTCTTCCCATCTCTCAGC | This Study |
| *SlPR1b1* | SlPR1b1-F | CTGGTGCTGGGGAGAATC | Kim et al. 2009 |
|  | SlPR1b1-R | GTCCGATCCAGTTGCCTACA | Kim et al. 2009 |
| *SlPR2* | SlPR2 qPCR-F | TGTAGACATGACGTTGATTGGCA | This Study |
|  | SlPR2 qPCR-R | ACGGAAGTGAAATCTGTCCTGG | This Study |
| *SlPR5* | SlPR5-qPCR-F | CAACATCCCTATGTCTTTCGGC | This Study |
|  | SlPR5-qPCR-R | AGGACCACATGGACCTTGAGTG | This Study |
| *SlACS2* | LeACS2-F | TCACCAACCAACACAACATCCAC | This Study |
|  | LeACS2-R | AACAAACTCGGAACCACCCTG | This Study |
| *AtActin* | AtActin F | GATGAGGCAGGTCCAGGAATC | This Study |
|  | AtActin R | GTTTGTCACACACAAGTGCATC | This Study |
