# Supplementary material for: The Tomato Leucine-Rich Repeat Receptor-Like Kinases SlSERK3A and SlSERK3B Have Overlapping Functions in Bacterial and Nematode Innate Immunity
Source: PLoS One. 2014 Mar 27;9(3):e93302. doi: 10.1371/journal.pone.0093302 (PMC3968124; doi:10.1371/journal.pone.0093302)
Supplement: Table S2 — List of primers used in cloning. (DOC) [file pone.0093302.s010.doc]

**Supplementary Table S2.** List of primers used in cloning

| **Gene** | **Primer name** | **Sequence (5'-3')*** |
| --- | --- | --- |
| *SlSERK3A* | SERK3A-VIGS-3'UTR-GatF | GGGGACAAGTTTGTACAAAAAAGCAGGCTGGGCCAAGATGATCTTTCATT |
|  | SERK3A-VIGS-3'UTR-GatR | GGGGACCACTTTGTACAAGAAAGCTGGGTCTGACACATATACCATTTAACCCC |
| *SlSERK3B* | SERK3B-VIGS-3'UTR-GatF | GGGGACAAGTTTGTACAAAAAAGCAGGCTGGCCAAGATGATCTTTCAGC |
|  | SERK3B-VIGS-3'UTR-GatR | GGGGACCACTTTGTACAAGAAAGCTGGGTATGTCATCAAAATTCCTGACATAC |
| *SlFLS2* | SlFLS2-VIGS GatF | GGGGACAAGTTTGTACAAAAAAGCAGGCTCCTCAAAGCTAAGTAAGATGGACTG |
|  | SlFlS2-VIGS GatR | GGGGACCACTTTGTACAAGAAAGCTGGGTGGAAACTTGGTGGTGGTTCAC |
| *SlSERK3A* | EcoRI-S3AKD F | CCGGAATTCCGGAGGAAACCACAAGACC |
|  | S3AKD-NotI R | ATAGTTTAGCGGCCGCTTATCATCTTGGCCCTGACAAC |
|  | SlS3A D418N-For | ATTATTCATCGTAACGTCAAAGC |
|  | SlS3A D418N-Rev | GCTTTGACGTTACGATGAATAAT |
| *SlSERK3B* | EcoRI-S3BKD F | CCGGAATTCCGAAGGAAACCGGAAGAC |
|  | S3BKD-NotI R | ATAGTTTAGCGGCCGCTTATCATCTTGGCCCTGATAAC |
|  | SlS3B D420N-Rev | GCTTTGACATTACGGTGGATG |
|  | SlS3B D420N-For | CATCCACCGTAATGTCAAAGC |
| *SlSERK3A* | SERK3A GatF 2nt | GGGGACAAGTTTGTACAAAAAAGCAGGCTCAATGGATCAGTCGGTGTTGG |
|  | SERK3A no stop 1nt GatR | GGGGACCACTTTGTACAAGAAAGCTGGGTGTCTTGGCCCTGACAACTCA |
|  | SERK3A GatR | GGGGACCACTTTGTACAAGAAAGCTGGGTTCATCTTGGCCCTGACAACT |
| *SlSERK3B* | SERK3B GatF 2nt | GGGGACAAGTTTGTACAAAAAAGCAGGCTCAATGATGGATCAATGGGTCTTG |
|  | SERK3B no stop 1nt GatR | GGGGACCACTTTGTACAAGAAAGCTGGGTGTCTTGGCCCTGATAACTCATC |
|  | SlS3B GatR with STOP | GGGGACCACTTTGTACAAGAAAGCTGGGTTCATCTTGGCCCTGATAACTC |
| *SlFLS2* | SlFLS2-GatF 2nt | GGGGACAAGTTTGTACAAAAAAGCAGGCTCAACAATGATGATGTTAAAGACAGTTG |
|  | SlFLS2-GatR 1nt noSTOP | GGGGACCACTTTGTACAAGAAAGCTGGGTGATCTTTTACCAAATGAGAAGG |
| *BAK1* | pBAK1 GatF | GGGGACAAGTTTGTACAAAAAAGCAGGCTGGATACAGACACAGATATGTCGTG |
|
|  | pBAK1r+S3Af | CCAACACCGACTGATCCATTTTATCCTCAAGAGATTAAAAACAAAC |
|  | pBAK1+S3Bf | CCAAGACCCATTGATCCATTTTATCCTCAAGAGATTAAAAACAAAC |
|

* Letters in red are part of Gateway recombination

* Letters in blue are additional nucleotides for inframe cloning in the vector
